# High relatedness of bioinformatic data and realistic experimental works on the potentials of *Fasciola hepatica* and *F. gigantica* cathepsin L1 as a diagnostic and vaccine antigen

**DOI:** 10.3389/fpubh.2022.1054502

**Published:** 2022-12-07

**Authors:** Ragab M. Fereig, Samy Metwally, El-Sayed El-Alfy, Hanan H. Abdelbaky, Obeid Shanab, Mosaab A. Omar, Abdullah F. Alsayeqh

**Affiliations:** ^1^Division of Internal Medicine, Department of Animal Medicine, Faculty of Veterinary Medicine, South Valley University, Qena, Egypt; ^2^Division of Infectious Diseases, Department of Animal Medicine, Faculty of Veterinary Medicine, Damanhour University, Damanhour, Egypt; ^3^Department of Parasitology, Faculty of Veterinary Medicine, Mansoura University, Mansoura, Egypt; ^4^Doctor of Veterinary Sciences, Veterinary Clinic, Veterinary Directorate, Qena, Egypt; ^5^Department of Biochemistry, Faculty of Veterinary Medicine, South Valley University, Qena, Egypt; ^6^Department of Parasitology, Faculty of Veterinary Medicine, South Valley University, Qena, Egypt; ^7^Department of Veterinary Medicine, College of Agriculture and Veterinary Medicine, Qassim University, Buraidah, Saudi Arabia

**Keywords:** *Fasciola hepatica*, antigen, diagnosis, vaccine, cathepsin L1

## Abstract

**Introduction:**

Fascioliasis is a parasitic foodborne disease caused by the liver flukes, *Fasciola hepatica* and *F. gigantica*. Such parasites cause serious illness in numerous domestic animals and also in humans. Following infection, the parasite secretes a variety of molecules that immediately interact with the host immunity to establish successful infection. These molecules include cathepsin L peptidase 1 (CatL1); the highly investigated diagnostic and vaccine antigens using various animal models. However, a few studies have analyzed the potentials of FhCatL1 as a diagnostic or vaccine antigen using bioinformatic tools and much less for FgCatL1. The present study provides inclusive and exclusive information on the physico-chemical, antigenic and immunogenic properties of *F. hepatica* cathepsin L1 (FhCatL1) protein using multiple bioinformatic analysis tools and several online web servers. Also, the validation of our employed available online servers was conducted against a huge collection of previously published studies focusing on the properties of FhCatL1as a diagnostic and vaccine antigen.

**Methods:**

For this purpose, the secondary, tertiary, and quaternary structure of FhCatL1 protein were also predicted and analyzed using the SWISS-MODEL server. Validation of the modeled structures was performed by Ramachandran plots. The antigenic epitopes of the protein were predicted by IEDB server.

**Results and discussion:**

Our findings revealed the low similarity of FhCatL1 with mammalian CatL1, lacking signal peptides or transmembrane domain, and the presence of 33 phosphorylation sites. Also, the containment of FhCatL1 for many topological, physico-chemical, immunological properties that favored its function of solubility and interaction with the immune components were reported. In addition, the earlier worldwide reports documented the high efficacy of FhCatL1 as a diagnostic and vaccine antigen in different animals. Altogether, FhCatL1 is considered an excellent candidate for using in commercialized diagnostic assays or vaccine products against fascioliasis in different animal species. Our assessment also included FgCatL1 and reported very similar findings and outputs to those of FhCatL1.

## Introduction

The liver flukes *Fasciola hepatica* and *F. gigantia* are common foodborne trematode parasites widespread worldwide. They affect a wide variety of host species causing fascioliasis. *Fasciola* parasites are major pathogens of ruminants and have a substantial impact on the health and wellbeing of animals. Severe infections can be fatal as a result of widespread liver damage caused by the migration of large numbers of young flukes. Chronic infections lead to anemia, weight loss and poor condition. However, low-grade infections are common and asymptomatic and are associated with slower growth and lower milk production (1, 2). People or animals become infected by *Fasciola* parasites *via* the ingestion of food contaminated with encysted metacercariae on aquatic plants or vegetables. According to the World Health Organization, several millions of people are at risk of fascioliasis and 2.4 million people in more than 70 countries are infected with this parasite (3).

Accurate diagnosis and vaccine development against virulent *Fasciola* spp. are a critical part of the control program of fascioliasis particularly with an increase in prevalence of liver flukes infection in human and animals. This elevation is predicted to aggravate because of climatic changes and emergence of triclabendazole-resistant *Fasciola* (4, 5). Usually, fascioliasis is diagnosed by coprology, e.g., sedimentation techniques. Coprology is easy to perform, but has a generally low sensitivity (30–70%), especially in cattle, and can only detect patent infections (6). Indirect enzyme-linked immunosorbent assay (ELISA) is another test for detection of *Fasciola* infections *via* screening for specific antibodies in serum and milk samples. Traditionally, these ELISAs utilize the crude fluke excretory/secretory (ES) proteins that possess strong antigenic characteristics. These tests demonstrated a high sensitivity (86.1–100 %) and specificity (70–99.3 %) (7). Among ES antigens, proteases are encountered in 73 % of the total protein content comprising cathepsin L, cathepsin B, serinoprotenases, metalloproteases, and legumains (8).

Cathepsin L1 (CL1) and CL2 are the most abundant of proteases in ES antigen and the two proteins accounting for 67 % and 28% of total cathepsin L proteins, respectively (9). However, CL1 has been the antigen of choice for diagnostic tests and as a potential vaccine candidate in numerous animal models (10, 11). In a more recent study, proteomic analyses revealed that the recombinant and native cathepsin L zymogens contain conserved, highly antigenic epitopes that are conformationally dependent. Additionally, the diagnostic capacities of cathepsin L zymogens were confirmed using serum and fecal panel of samples, suggesting an excellent efficacy as markers of infection and for monitoring treatment efficacy (12).

Bioinformatics is one of the most current study fields to better address and monitor biological challenges (13). It is frequently used to evaluate the expression of genes and proteins as well as to forecast their general properties such as antigenicity and immunogenicity. These factors will significantly increase our understanding of these molecules and help scientists choose the best proteins or particular epitopes for studies on vaccines or medical diagnosis.

Epitopes are typically identified by showing that certain peptide fragments and certain proteins have antigenic reactivity. Since the accessibility, hydrophilicity, and mobility of polypeptide chain segments have been linked to the position of epitopes in proteins, it is conceivable to infer from the primary structure, in which linear peptides are most likely to correspond to those epitopes (14). In order to gain some insights into the molecular structure of its insoluble counterpart, secondary structure and antigenicity predictive methods have been applied to the amino acid sequences of different origins (15). This indicates that such a mechanism can be applied for diagnostic or immunogenic applications, such as our topic. It has been tried numerous times to infer the location of antigenic sites in proteins from specific traits of their primary, secondary, or tertiary structures. According to recent findings, the antigenicity definition varies depending on the type of probe employed to analyze it (16). Without knowledge of the protein's tertiary structure or the presence of discontinuous epitopes, the specificity of an immune response might also be accurately predicted for vast protein sections. Instead, antigenicity is a local attribute of the protein sequences, and the immune response's specificity and antigenicity are determined by the protein sequences' composition, secondary structure, solvent accessibility, and evolutionary conservation (17). Due to its continuous epitope distribution, we propose that the tertiary protein structure may have the same antigenicity as the primary structure. A linear peptide fragment of the epitope is highly cross-reactive with the matching antibodies. These epitopes are single-stretch polypeptide chains that are referred to as linear or continuous epitopes (18). By applying additional features, the B-cell epitope prediction task can be further enhanced. For instance, understanding the secondary structure and solvent accessibility of proteins in their three-dimensional (3D) form is essential. All of the evidence points of the efficient antigenicity being shared by primary and tertiary protein structures.

Few studies have analyzed the potentials of FhCatL1 as diagnostic or vaccine antigens using bioinformatic tools and much less for FgCatL1 (19, 20). Nevertheless, such studies have only focused on certain properties and using few analysis tools. Thus, the present study is providing inclusive and exclusive information on the physico-chemical, antigenic and immunogenic properties of *F. hepatica* cathepsin L1 (FhCatL1) protein using multiple bioinformatic analysis tools and several online web servers. In addition, data of bioinformatic analyses have been compared with the previous heritage of available studies investigated the potentials of FhCatL1and FgCatL1in immnunodiagnostic and vaccine researches.

## Materials and methods

### Amino acid sequence retrieval

The amino acid sequence for *F. hepatica* cathepsin L1 (FhCatL1) was obtained in FASTA format from the GenBank (AJ279092.1) database at https://www.ncbi.nlm.nih.gov. Then, using the corresponding cathepsin L1 proteins from FhCatL1, as well as those from *F. gigantica* (JQ342985.1), humans (BC142983.1), and cattle, multiple sequence alignment was performed using Clustal Omega (https://www.ebi.ac.uk/Tools/msa/clustalo/) (BC134741.1). For full matching with FhCatL1, first 15 amino acid (MRLFILAVLTVGVLG) was deleted from our analyzed *F. gigantica* (JQ342985.1).

### Post-translational modification (PTM) sites and transmembrane domains

To identify the signal peptide in the sequence, the SignalP online tool (https://services.healthtech.dtu.dk/service.php?SignalP-5.0) was utilized. A Sec signal peptide (Sec/SPI), a Lipoprotein signal peptide (Sec/SPII), a Tat signal peptide (Tat/SPI), or no signal peptide at all can be found in the protein (Other). Additionally, using the TMHMM 2.0 online server (https://services.healthtech.dtu.dk/service.php?TMHMM-2.0), putative transmembrane domains of FhCatL1 and FgCatL1 were predicted (21). Using the online isoelectric point calculator at http://isoelectric.org, the isoelectric point (pI) and molecular weight of FhCatL1 and FgCatL1 was calculated. The molecular weight and diameter of the selected amino acids from FhCatL1 and FgCatL1of number 311 were determined.

### Physical, chemical and morphometric characteristics

The NetPhos 3.1 server (https://services.healthtech.dtu.dk/service.php?NetPhos-3.1) used ensembles of neural networks to show the locations of phosphorylation sites for serine, threonine, and tyrosine. Two web servers were used to clarify the FhCatL1 and FgCatL1 secondary structure, including the Garnier-Osguthorpe-Robson (GOR IV) servers (https://npsa-prabi.ibcp.fr/cgi-bin/npsa_automat.pl?page=/NPSA/npsa_gor4.html) and position specific iterated prediction (PSIPRED) (22, 23), based on PSI-BLAST results (http://bioinf.cs.ucl.ac.uk/psipred). Then, three-dimensional (3D) protein models were created using SWISS-MODEL, a fully automated online server for protein structure homology modeling that may be found at (https://swissmodel.expasy.org/) (24). Using the Ramachandran plot analysis used by the Swiss model, the tertiary structure was further refined and validated for both FhCatL1 and FgCatL1. This was done based on the percentage of residues in the outlier and preferred regions. Additionally, using the ProtScale tool (https://web.expasy.org/protscale/), graphical representations of linear epitopes were created based on average flexibility, beta-turn, alpha-helix, hydrophobicity, and the percentage of accessible residues and molecular weight (25).

### Prediction of B-cell epitopes, antigenicity, and hydrophilicity

Based on the physicochemical characteristics of amino acids including hydrophilicity (26), and antigenicity (27), the Immune Epitope Database (IEDB) (http://tools.immuneepitope.org/bcell/) was employed by the IEDB server to predict the B-cell epitopes. ElliPro predictor was used to analyze linear and conformational (discontinuous) epitopes based on 3D structures prediction of FhCatL1. Crystal Structure of ProCathepsin L1 from *Fasciola hepatica* (206X) that showed high similarity with our target sequence of FhCatL1, as it is available as PDB file, the required input data for ElliPro searches was used for further analysis of FhCatL1 (http://tools.iedb.org/ellipro/). Similarly, Disco Tope tool that can predict the conformational antibody epitope via 3D structures was used employing the same the protein model 206X (http://tools.iedb.org/discotope/). A summary for the predictor servers used in the current study for analyses of different properties of FhCatL1 was illustrated in Table 1.

**Table 1:** Predictors used to analyze different features of FhCatL1 protein.

**Item**	**Characteristics**	**Predictor**	**Description**
Molecular properties	Molecular weight	IPC	Amino acids weights
	Diameter	IPC	Calculated from empirical results of several model proteins and enzymes.
	Isoelectric point	IPC	Amino acids pK values
Peculiarities in the protein sequence	Multiple sequence alignment	CLUSTAL OMEGA	Comparison of similarity of input target of amino acid sequence with other sequence manually selected.
			
	Signal peptide cleavage sites	SignalP 5.0	Prediction of cleavage sites and a signal peptide/non-signal peptide prediction based on a combination of several ANN
	Phosphorylation sites	NetPhos 3.1	Predicts serine, threonine or tyrosine phosphorylation sites in proteins using ensembles of neural networks.
Secondary and tertiary structures	Transmembrane helices in proteins	TMHMM 2.0c	Membrane protein topology prediction method based on a HMM
	Secondary structure	PSIPRED	Provides depiction for regions of disorder and transmembrane helix packing; contact analysis; fold recognition; structure modeling; and prediction of domains and function.
		GOR IV	Predicts locations of alpha-helix and beta-strand from amino acid sequence.
	Tertiary structure	ExPasy Swiss model	Subsequently, three-dimensional (3D) protein models were constructed using a fully automated protein structure homology-modeling
		Ramachandran Plots	Protein structure refinement and validation by visualizing energetically allowed regions for backbone dihedral angles ψ against ϕ of amino acid residues in protein structure.
		ExPasy Protscale model	An amino acid scale is defined by a numerical value assigned to each type of amino acid. The most frequently used scales are the hydrophobicity or hydrophilicity scales and the secondary structure conformational parameters scales, and others.
Quaternary structure		SWISS Model	
Immune stimulation	B-cell epitopes	Bepipred Linear Epitope Prediction 2.0	Predicts B-cell epitopes from a protein sequence, using a Random Forest algorithm trained on epitopes and non-epitope amino acids determined from crystal structures
		ElliPro	Predicts linear and discontinuous antibody epitopes based on a protein antigen's 3D structure. PDB format was used as an input protein structure (206X).
		Disco Tope	Predicts discontinuous epitopes from 3D structures of proteins in PDB format
	Antigenicity	Kolaskar and Tongaonkar antigenicity scale	A semi-empirical method which makes use of physicochemical properties of amino acid residues and their frequencies of occurrence in experimentally known segmental epitopes was developed to predict antigenic determinants on proteins.
Other properties	Hydrophilicity	Parker hydrophilicity prediction	Hydrophilic scale based on peptide retention times during high-performance liquid chromatography (HPLC) on a reversed-phase column was constructed

## Results and discussion

### Multiple sequence alignment and molecular weight

The amino acid sequence of FhCatL1 protein was obtained from GenBank accession ID: AJ279092.1which was submitted by Cornelissen et al. (28). Then, CLUSTALO-based multiple sequence alignment of FhCatL1 was applied against cathepsin L1 of *F. gigantica*, as well as human and cattle amino acids as examples for highly susceptible host species for fascioliasis. Similarity was 94.2, 44.4, and 40.2% for cathepsin L1 of *F. gigantica*, human and cattle, respectively (Supplementary Figures S1A,B). These results demonstrated the high similarity of FhCatL1 with other *Fasciola* cathepsin L proteins and high differences with those of human or cattle suggesting the low probability of cross reaction with samples of such animal hosts.

### Signal peptide, transmembrane domains, and isoelectric point

Based on the SignalP-5.0 and TMHMM output, no signal peptides or transmembrane domain were found for FhCatL1 (Supplementary Figures S2A–D). Our results based on transmembrane domain analysis suggest the cytoplasmic localization of FhCatL1 and FgCatL1. Cytosolic proteins include large complexes of enzymes that are responsible for several metabolic pathways, protein biosynthesis, and other important cell-signaling processes. Indeed, nuclear or cytoplasmic protein extracts are important for various applications, including RNA binding, *in vitro* transcription, mRNA splicing, or gene-expression studies. Thus, the antibody targeted cytosolic protein can serve as a molecular tool to enhance our knowledge of protein–protein interactions between *Fasciola* parasites and the host cell machinery (29). The theoretical average of pI of FhCatL1 was 5.2. Using all predictors, the pI was not lower than 5 except for Patrickios; pI = 3.248. In addition, the theoretical average of pI of FgCatL1 was 5.11 and Patrickios pI was 2.93 (Supplementary Figures S3A,B). The amino acids sequences of FhCatL1 protein include 311 amino acid residues with an estimated molecular weight of 35.2 kDa (antigens which have MW of < 5–10 KDa are considered as weak immunogens) **(****30****)**, with a diameter equal to 4.67487 nanometer (nm). Similarly, FgCatL1 (JQ342985.1) consists of 311 amino acids with estimated MW of 35.2 kDa with similar diameter to FhCatL1 (Supplementary Figures S3C,D).

Two major components are affecting the production level of the recombinant protein; the presence of signal peptides and secretory machinery. The signal sequence is often a short peptide that carries information about the protein target and secretion pathway zip codes at the N-terminus of the most recently made proteins. Additionally, the antigen presentation would be improved by the absence of any potential transmembrane domains in order to stimulate humoral and cell-mediated immunity as well as a quick response (21, 31). A number of analytical biochemistry and proteomics procedures, such as 2-D polyacrylamide gel electrophoresis or capillary isoelectric focusing used in conjunction with high-throughput mass spectrometry, depend on the accurate estimate of the isoelectric point (pI) through the amino acid sequence. Additionally, protein crystallization experiments can benefit from pI estimation (32).

### Physico-chemical and morphometric characteristics

**Figure 1:**
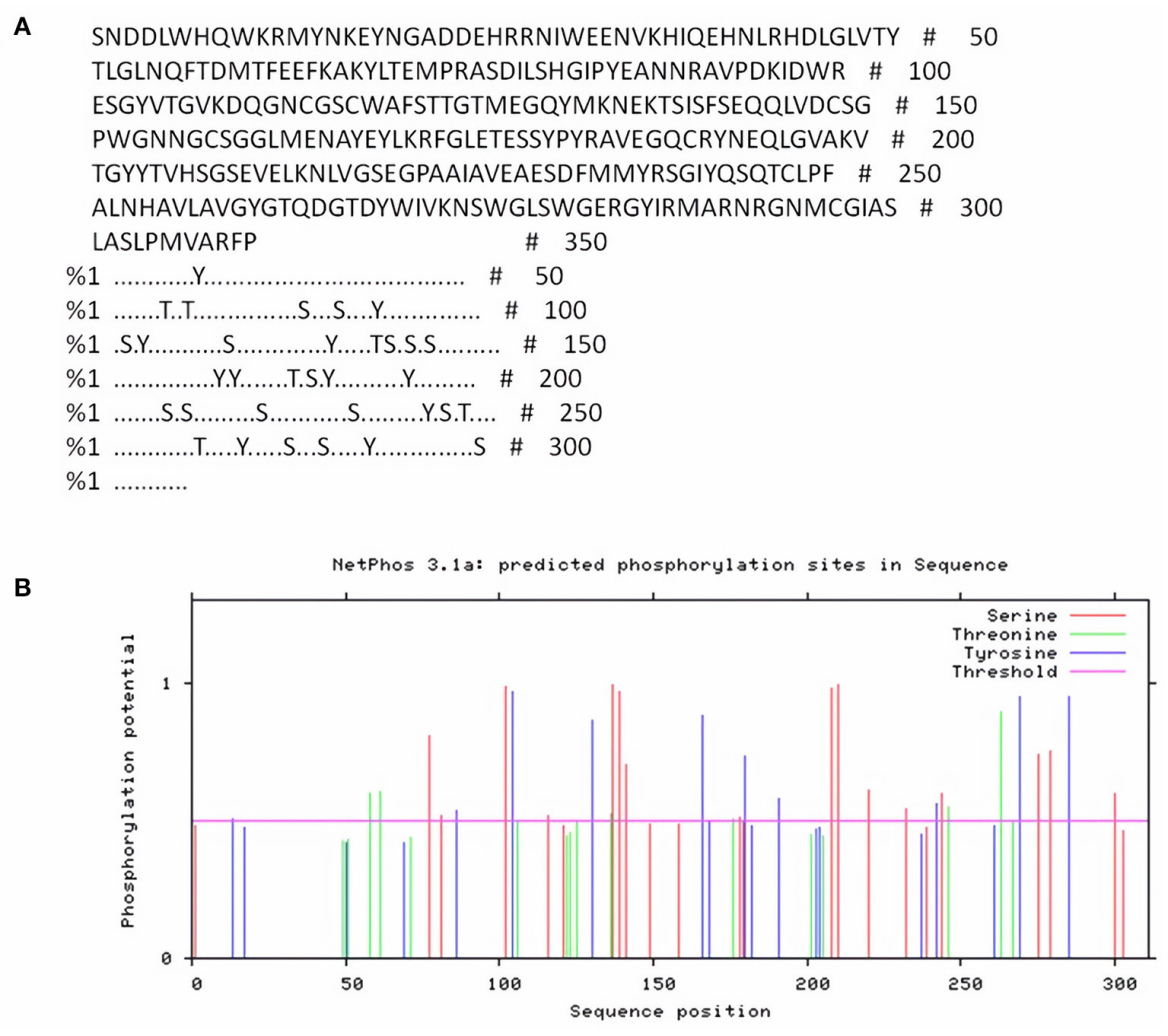
NetPhos server output for FhCatL1 phosphorylation sites. **(A)** The number of predicted sites, based on S (serine), T (threonine) and Y (tyrosine); **(B)** Prediction diagram of FhCatL1 phosphorylation sites (https://services.healthtech.dtu.dk/service.php?NetPhos-3.1).

NetPhos server was used for detection of phosphorylation sites in FhCatL1 protein based on serine, threonine, and tyrosine amino acids. Analysis results revealed that there were 33 phosphorylation sites in the FhCatL1 protein sequence, with 16 serine (S), six threonine (T), and eleven tyrosine (Y) as shown in Figures 1A,B. Consistently, analysis of FgCatL1 revealed 33 phosphorylation sites with 16 serine (S), 3 threonine (T), and 14 tyrosine (Y) (Supplementary Figure S4). The existence of phosphorylation sites indicates the high liability of protein for post translational modifications (PTMs) processes. PTMs in eukaryotic cells, such as the cells of parasites, are significant for choosing the correct expression system in the development of recombinant proteins and might affect the biological function of the protein (33, 34).

The FhCatL1 protein contained three main secondary structure components, consisting of 53.38 % random coil, 24.44 % extended strand, and 22.19 % alpha helix, as shown by PSIPRED and GOR IV servers. For FgCatL1 amino acid sequences encompasses 52.41 % random coil, 25.08 % extended strand, and 22.51 % α-helix in secondary structure (Supplementary Figures S5, S6). Proteins' primary biological behavior is primarily determined by their spatial organization. The sequence of hydrogen bonding between carboxyl oxygen and amino hydrogen atoms in a polypeptide chain, with α-helices and 2D-structures being the most frequent types, typically determines the secondary structure (35).

**Figure 2:**
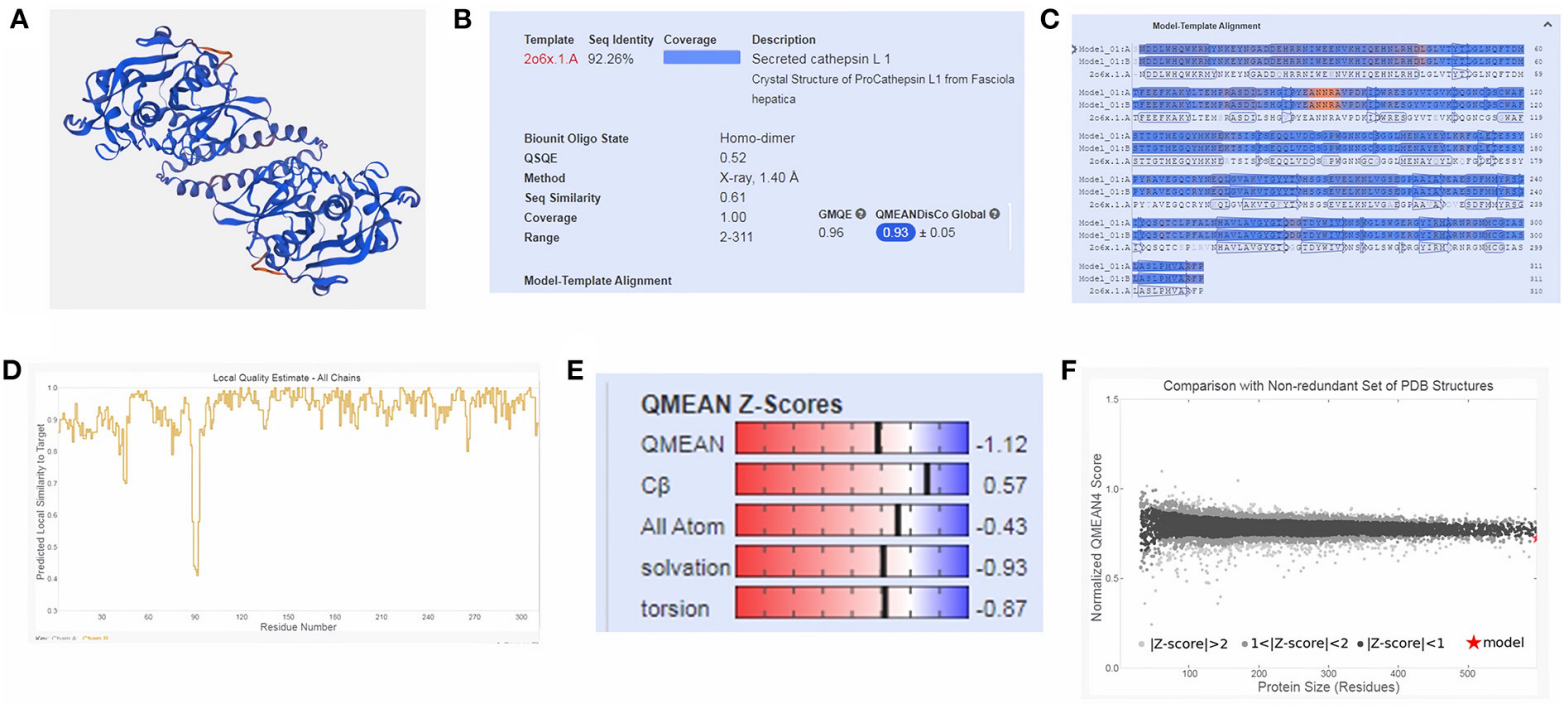
Tertiary structure of FhCatL1 using SWISS-MODEL server. SWISS-MODEL server results of **(A)** Computed three-dimensional model; **(B)** Sequence identity and coverage data; **(C)** Model-template alignment; **(D)** Local quality estimate; **(E)** Global quality estimate; **(F)** Comparison with non-redundant set of PDB structures (https://swissmodel.expasy.org/).

Moreover, three-dimensional (3D) protein models were constructed for FhCatL1and FgCatL1using a fully automated protein structure homology-modeling web server called SWISS-MODEL. In total, 50 SWISS-MODEL templates were made for FhCatL1 protein sequence, among which a single model highly matching our target was built. The model showed highest coverage and 92.26% sequence identity. Global model evaluation GMQE and QMEANDisCo of any analyzed model gives an overall model quality measurement between 0 and 1, with higher numbers indicating higher expected quality. GMQE and QMEANDisCo in our model was 0.96 and 0.93, respectively, suggesting high quality of expected model measurement and assessment (Figures 2A–D). The significance of the QMEAN Z-score as an indicator of protein stability was further highlighted by the fact that our model had significantly high QMEAN Z-scores in a pairwise comparison with their homologous mesophilic counterparts. In our case, QMEAN *Z*-score was −1.12 which is close to zero suggesting high accuracy and reliability of protein modeling (Figure 2E). In addition, our target model was distributed in the same area when compared with non-redundant set of protein data base reference structures (Figure 2F). Red color in sequence alignment and amino acid sequence ANNRA, and low indentation at low quality estimate curve near 0.4 are showing area of instability which might be prone to mutation. Three dimensional structures was also applied for FgCatL1 and found similar data and findings to FgCatL1. Crystal Structure of ProCathepsin L1 from *Fasciola hepatica* was found and used as a model for further template to FgCatL1 (Supplementary Figure S7).

**Figure 3:**
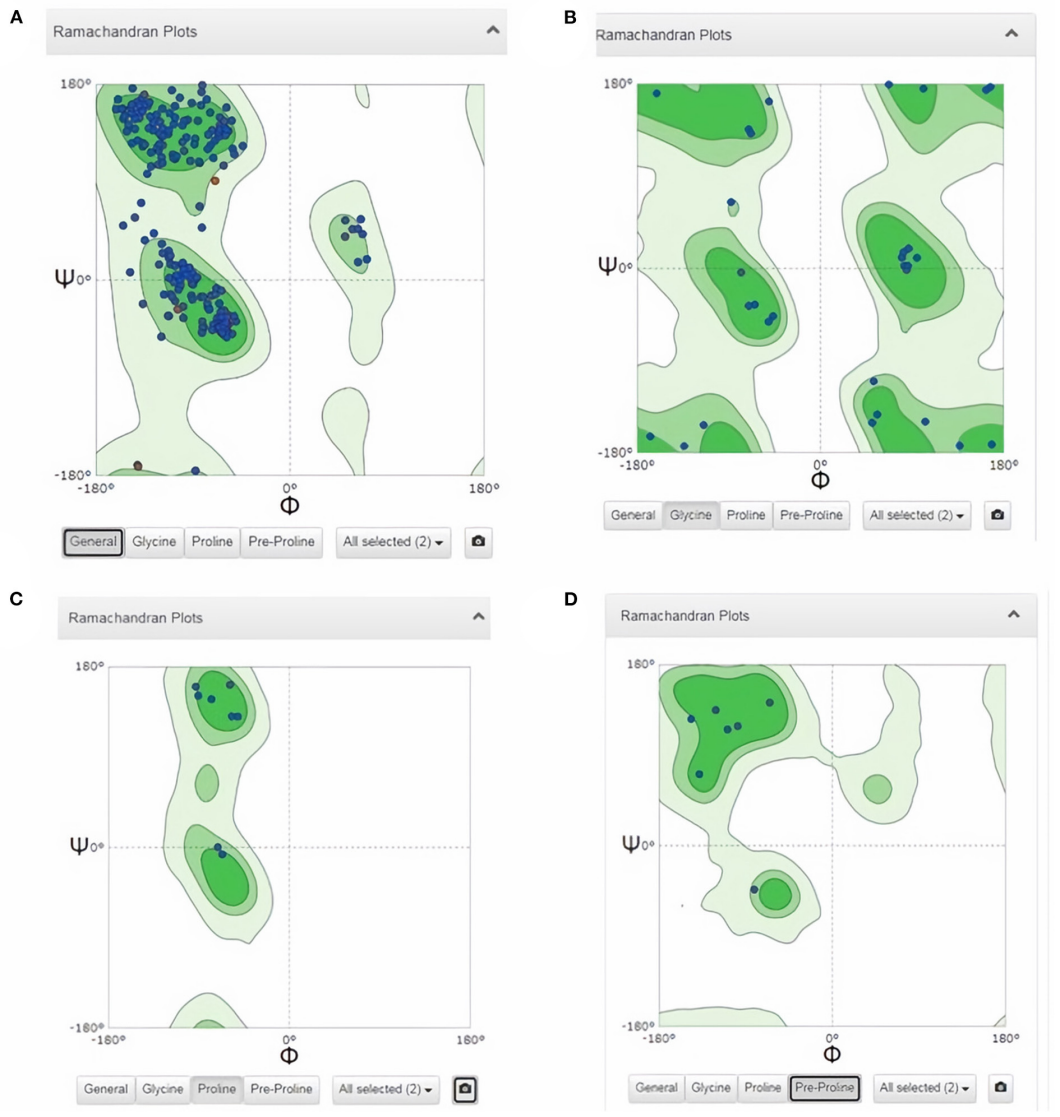
Analysis of 3D model of FhCatL1 *via* Ramachandran plot from SWISS-MODEL server. The model demonstrated MolProbity (0.92), Clash score (0.21), Poor rotamers (1.0) and Ramachandran favored (95.78%), no Ramachandran outliers, C-Beta deviations or bad bonds, Rotamer outliers (1.16%), bad angels (30/6856) and twisted proline (2/18). **(A)** General plot, **(B)** Glycine based plot, **(C)** Proline based plot, **(D)** PreProline based plot (https://swissmodel.expasy.org/).

**Figure 4:**
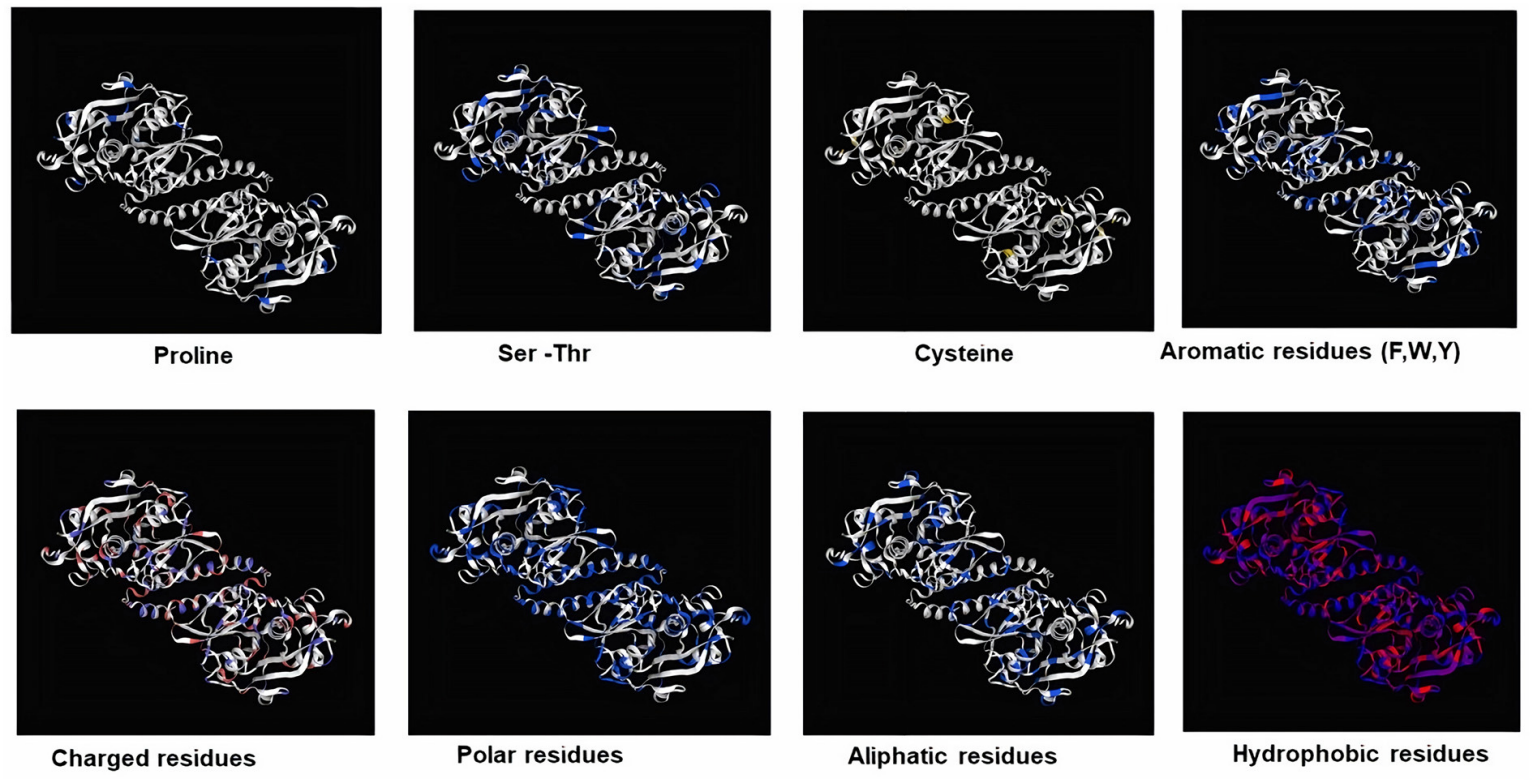
Mapping of different structure of amino acid residues in 3D structure model of FhCatL1 (https://swissmodel.expasy.org/).

**Figure 5:**
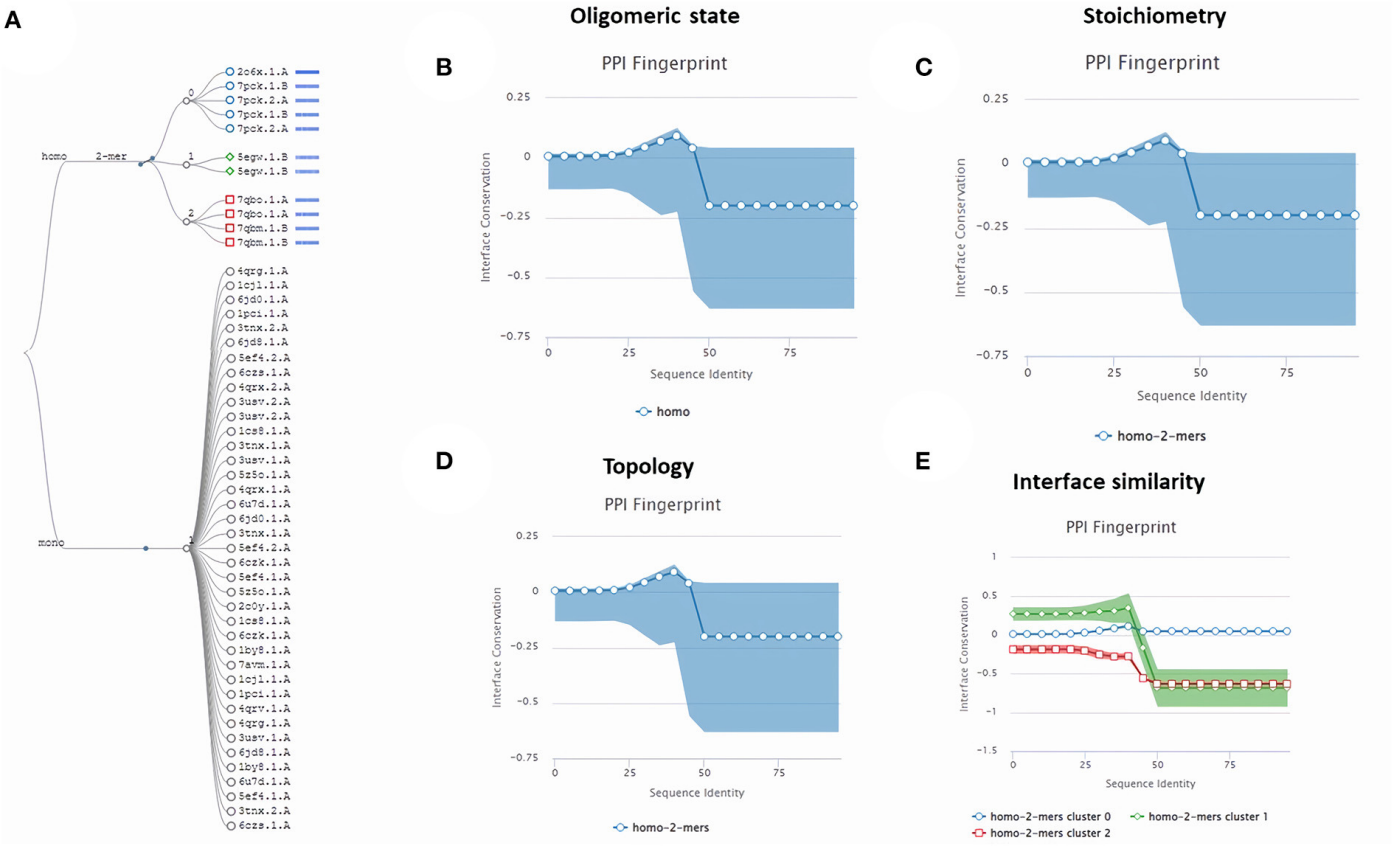
Quaternary structure analysis of FhCatL1 as a homo-dimer model **(A)** Structural clustering tree of FhCatL1 homologs with known structure. Each leaf is a template labeled with the PDB code and a bar indicating sequence identity and coverage (darker shades of blue refer to higher sequence identity). The decision tree follows the described levels of clustering: oligomeric state **(B)**, stoichiometry **(C)**, the topology of the complexes **(D)**, and interface similarity **(E)**. The PPI fingerprint curves of our oligo-dimeric target shows high consistency with PDB model code 206X with high consistency as shown oligomeric state, stoichiometry and topological characteristics. Also, our homo-dimer target showed a higher similarity for interface interactions with cluster 0 (blue circle) included 206X than cluster 1 (green rhomboid) and 2 (red square).

**Figure 6:**
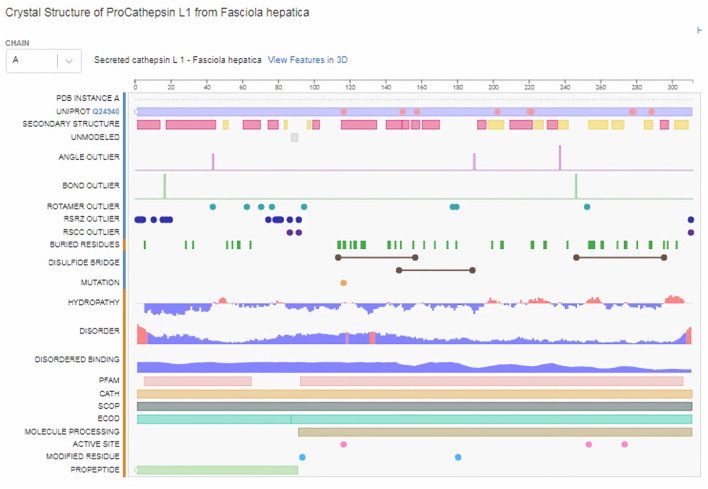
Structural and physical properties of FhCatL1 protein from protein data base (PDB).

Subsequently, the chosen 3D model was analyzed *via* Ramachandran plot from SWISS-MODEL server. The model demonstrated MolProbity (0.92), Clash score (0.21), Poor rotamers (1.0) and Ramachandran favored (95.78%), no Ramachandran outliers, C-Beta deviations or bad bonds, Rotamer outliers (1.16%), bad angels (30 / 6856) and twisted proline (2/18) (Figure 3). These results indicate the high quality of 3D model of FhCatL1. Also, Ramachandran plot was applied for FgCatL1 revealed a very similar data to those obtained for FhCatL1 (Supplementary Figure S8). As such, to comprehend the influences between both structures and functions, assessment of 3D structure is the key aim of expecting a protein's nature (36, 37). More accurate mapping of different structure of amino acid residues in our 3D structure model was also analyzed (Figure 4). Furthermore, analysis of such model was conducted *via* quaternary structure either for degree of oligomerization, stoichiometry, topology, and the interface similarity (Figure 5). Our target sequence showed a homo-dimeric oligo state. High sequence similarity was observed when our target sequence of FhCatL1 was compared against conserved model Crystal Structure of ProCathepsin L1 from *Fasciola hepatica* (206X) at SWISS-MODEL repository for all tested characters of oligomerization, stoichiometry, topology, and the interface similarity based on quaternary structure analysis (Figures 5A–E). The dimeric forms of FhCatL1 have a stronger interface conservation signal with clade including 206X model with high similarity to our target sequence. This stronger conservation is observable using different evolutionary distance thresholds. Such knowledge about stoichiometry, topology, and the interface similarity aid in the understanding of FhCatL1 interactions with other biomolecules, and provide insights into their function in various biochemical processes, such as signal transduction, immune response or metabolism (38). As 206X sequence data is already publicly available at http://oligo.swissmodel.expasy.org, and at https://www.rcsb.org/sequence/2O6X, similar data can be obtained from these websites. However, our collection from other servers will assist the molecular biologists and biochemists by providing a detailed description and discussion for the physico-chemical properties of FhCatL1 protein. SWISS MODEL provides an automated tool for analysis of a protein sequence using different levels of structural forms particularly tertiary and quaternary structures (24). Further results regarding structural and physical properties were also obtained from protein data bank (PDB) after searching FhCatL1 protein (Figure 6). These results confirmed those of low values for outlier amino acid residues and additionally reported minimum liability for hydropathy, mutation and disorders. Recombinant proteins increasingly replace complete organisms in current immunotherapy and vaccination in order to stimulate therapeutic or protective immune responses. Protein structural stability is essential for the effective presentation of antigenic peptides on MHC, which is critical for inciting powerful immune responses (39). Thus, our data concerning the physical and chimerical properties, suggest the usefulness of FhCatL1 for further immunological investigations. A summary of physico-chemical and structural properties is shown in Supplementary Table S1.

### Immune-epitope analyses of FhCatL-1

**Figure 7:**
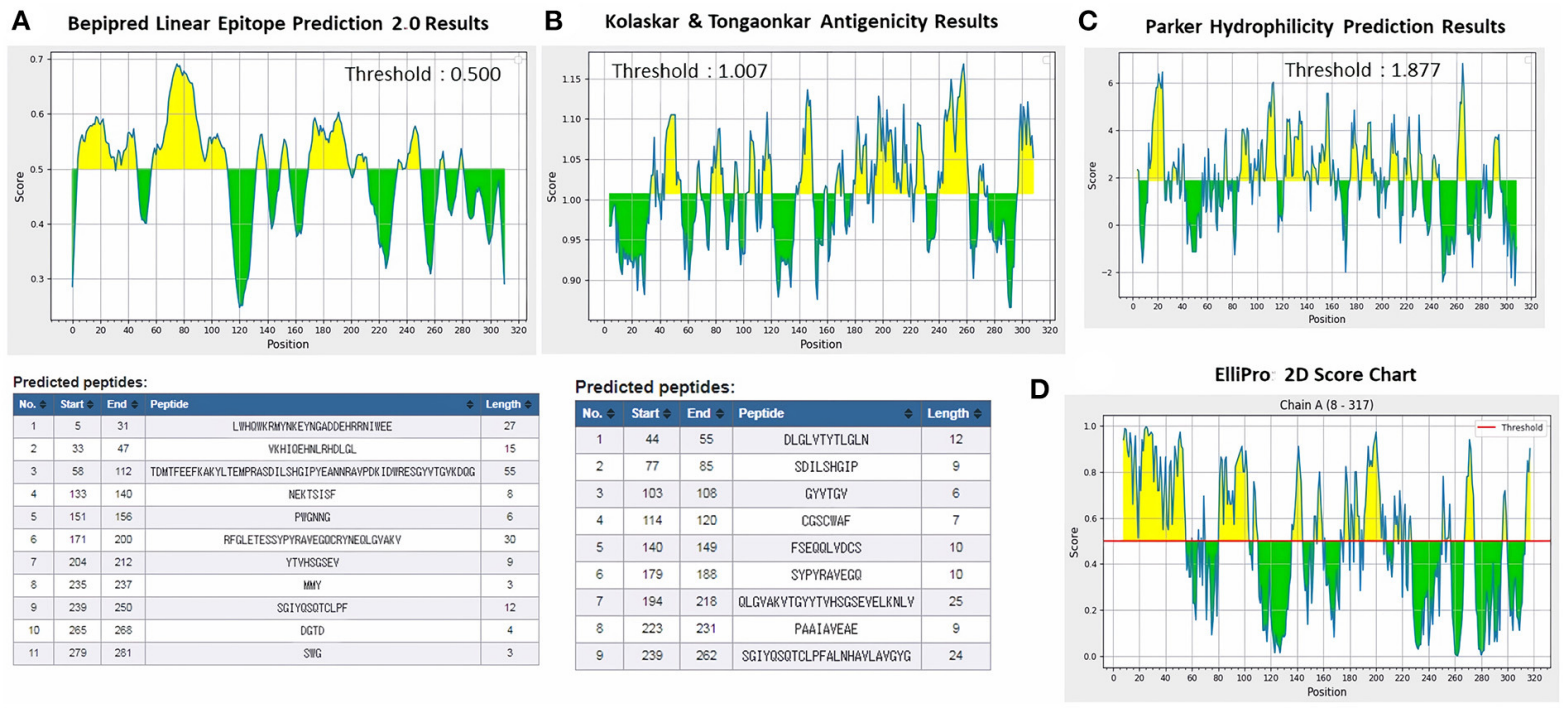
Immunogenicity, antigenicity, and hydrophilicity of FhCatL1 protein. **(A)** Bepipred linear epitopes prediction; **(B)** Antigenicity; **(C)** Hydrophilicity; **(D)** ElliPro: 2D Score Chart epitope prediction. *x*-axis and *y*-axis represent position and score, respectively. The horizontal line indicates the threshold or the average score. Yellow colors (above the threshold) indicate favorable regions related to the properties of interest. Green color (below the threshold) indicates the unfavorable regions related to the properties of interest. The results were analyzed by the Immune Epitope Database (IEDB) (http://tools.immuneepitope.org/bcell/).

**Figure 8:**
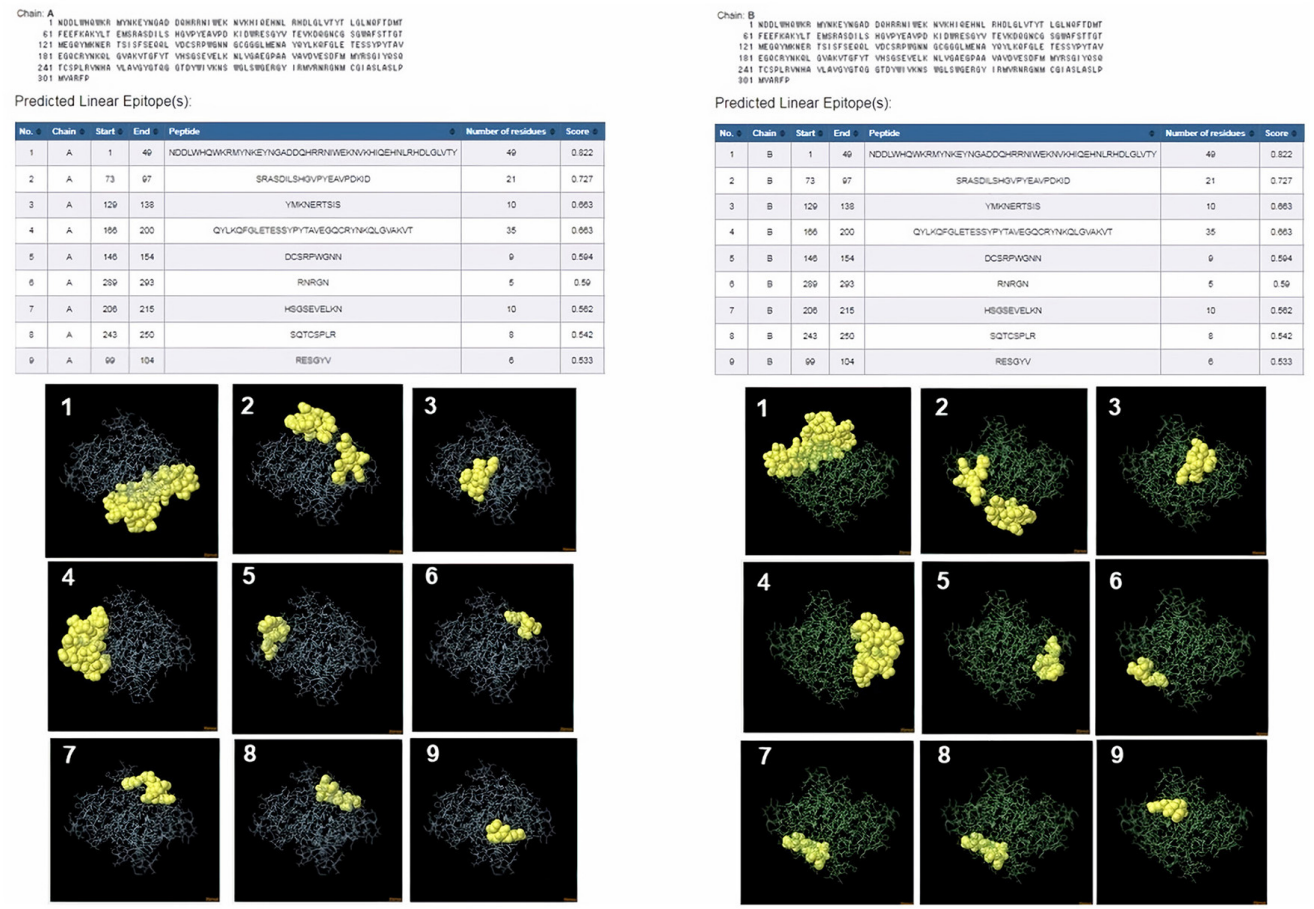
ElliPro: Linear antibody epitope 3D structures prediction of FhCatL1. Crystal Structure of ProCathepsin L1 from *Fasciola hepatica* (206X.1A) chain A PDB file was used as input data for ElliPro searches. Input sequence, table of predicted epitopes, and Jmol visualization of the 9 predicted epitopes shows the epitope residues are in yellow and the rest of the protein is in violet **(left panel)**. Crystal Structure of ProCathepsin L1 from *Fasciola hepatica* (206X.1B) chain B PDB file was used as input data for ElliPro searches. Input sequence, table of predicted epitopes, and Jmol visualization of the 9 predicted epitopes shows the epitope residues are in yellow and the rest of the protein is in green **(right panel)** (http://tools.iedb.org/ellipro/).

**Figure 9:**
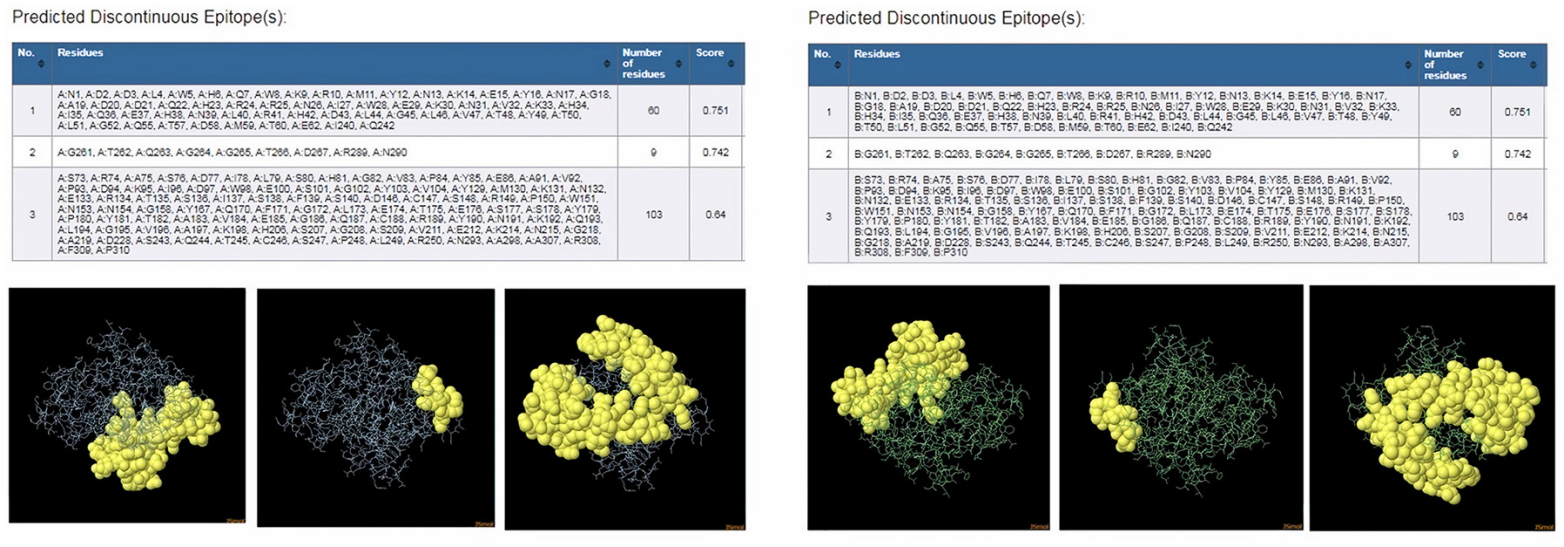
ElliPro: Discontinuous (conformational) antibody epitope 3D structures prediction of FhCatL1. Crystal Structure of ProCathepsin L1 from *Fasciola hepatica* (206X.1A) chain A PDB file was used as input data for ElliPro searches. Table of predicted epitopes, and Jmol visualization of the 3 predicted epitopes shows the epitope residues are in yellow and the rest of the protein is in violet **(left panel)**. Crystal Structure of ProCathepsin L1 from *Fasciola hepatica* (206X.1B) chain B PDB file was used as input data for ElliPro searches. Table of predicted epitopes, and Jmol visualization of the 3 predicted epitopes shows the epitope residues are in yellow and the rest of the protein is in green **(right panel)** (http://tools.iedb.org/ellipro/).

**Figure 10:**
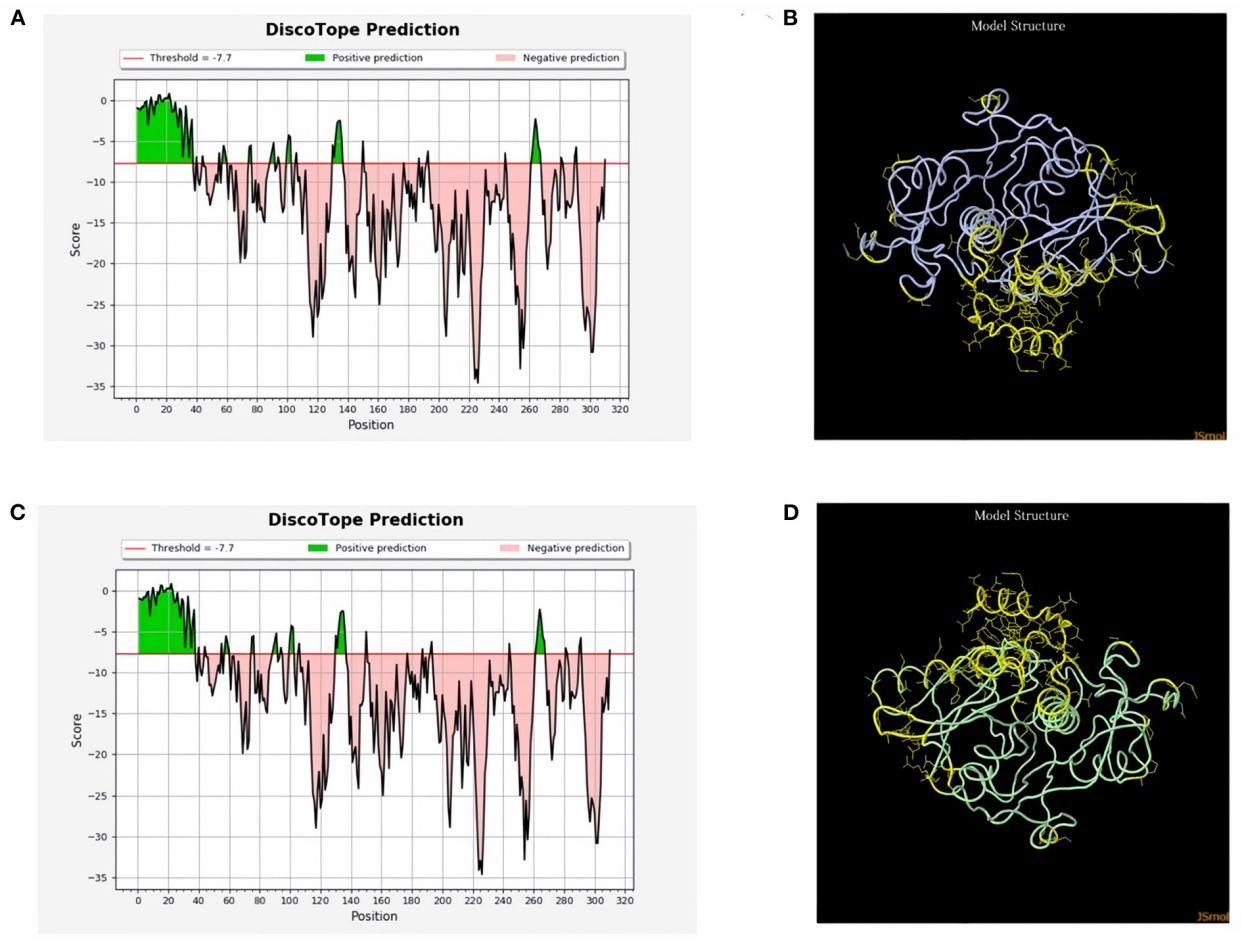
Disco Tope: Discontinuous (conformational) antibody epitope 3D structures prediction of FhCatL1. Crystal Structure of ProCathepsin L1 from *Fasciola hepatica* (206X). Chain A PDB file was used as input data for Disco Tope searches (https://www.rcsb.org/structure/2O6X) for visualization of 2 D chart **(A)** and Jmol visualization **(B)** of the predicted epitope. Chain B PDB file was used also as input data for visualization of 2 D chart **(C)** and Jmol visualization **(D)** of the predicted epitope shows the epitope residues are in yellow and the rest of the protein is in violet (http://tools.iedb.org/discotope/).

ProtScale online tool was applied to predict linear B-cell epitopes for FhCatL1 and FgCatL1 according to hydrophobicity, alpha-helix, beta-turn, average flexibility, percent of accessible residue, and molecular weight (Supplementary Figure S10). Strong signals were obtained in all the plots of different scales by most amino acids residues suggesting the high presence of immune epitopes in our tested sequence of FhCatL1 and FgCatL1. High values estimated for epitopic locations with good hydrophilicity and potent antigenicity were found using the IEDB server. Bepipred Linear Epitope Prediction 2.0 predicted 11 anticipated peptides and a threshold of 0.5, with estimated lengths ranging from 3 to 55 amino acids for the linear B-cell epitope areas (Figure 7A). Kolaskar and Tongaonkar also predicted nine anticipated peptides with high antigenicity indices. Antigenicity with a threshold of 1.007, and an estimated amino acid range of 6 to 24 was observed (Figure 7B). Also, the hydrophilicity of FhCatL1 was detected by Parker Hydrophilicity Prediction scale showing high index of water solubility with threshold 1.877 (Figure 7C). Similar results were obtained when analyzing sequence of FgCatL1 using before mentioned predictors (Supplementary Figure S11). In addition, linear and discontinuous antibody epitopes based on a protein antigen's 3D structure was analyzed using ElliPro. This analysis server based on utilizing the PDB file of a certain protein not amino acids sequence as input data. Thus, the available protein file in PDB named 206X was used to assess the different epitopes of FhCatL1. As a primary analysis, ElliPro 2D chart was also illustrated which showed high prediction of epitope residues (Figure 7D). For epitope mapping, ElliPro 3D analysis tool was used to reveal the linear and conformational epitopes. For this purpose, chain A and B of the used PDB file of 206X.1A and 206.1B was assessed separately, respectively for more accurate prediction. ElliPro search revealed 9 predicted epitopes with showing 3D structure and Jmol visualization. The largest residues of the used protein model consist of linear epitopes with 21 to 49 amino acids with score ranged from 0.663 to 0.822 (Figure 8). Regarding the conformational epitopes, ElliPro analysis demonstrated 3 amino acid residues with score ranged from 0.64 to 0.751 (Figure 9). Disco Tope is another analysis tool used to predict the conformational antibody epitope based on 3D structures prediction and using PDB file as input data. Crystal Structure of ProCathepsin L1 from *Fasciola hepatica* (206X.1A or 206X.1B) was assessed followed by visualization of 2D charts and Jmol visualization of the predicted epitopes. As shown in Figure 10, 2D chart and Jmol display the structure with high positive predictions (74 residues from total 310) highlighted in yellow in both panels. These results revealed the high potentials of FhCatL1 for interacting with immune cells and generating specific antibodies.

The interaction between antibodies and antigens is one of the most important immune system mechanisms for clearing infectious organisms from the host. Antibodies bind to antigens at sites referred to as B-cell epitopes. Identification of the exact location of B-cell epitopes is essential in several biomedical applications such as; rational vaccine design, development of disease diagnostics and immunotherapeutics. However, experimental mapping of epitopes is resource intensive making *in silico* methods an appealing complementary approach (40). An epitope for a protein antigen may be a brief peptide from the protein sequence, known as a linear epitope, or it may be a patch of atoms on the surface of the protein, known as a discontinuous or conformational epitope. The goal of discontinuous epitope prediction is to create a molecule that can mimic the structure and immunogenic properties of an epitope and replace it either in the process of antibody production—in this case, an epitope mimic can be thought of as a prophylactic or therapeutic vaccine—or antibody detection in medical diagnostics or experimental research. While, linear epitopes can be directly used for the design of vaccines and immunodiagnostics (41, 42).

Herein, we provided essential information on the physico-chemical, antigenic, and immunogenic capacities of FhCatL1 and much less extent of FgCatL1. This analysis was variably relying on the availability of data in three major data set repositories including NCBI, SWISS-MODEL, and PDB. However, the data presented here is providing numerous beneficial insights on the characterization of FgCatL1 from different aspects ranged from structural to advanced antigenic or immunogenic properties. Nevertheless, these data have the limitations of generation using only bioinformatic or computed analysis. Thus, in the following section, a literature review was conducted to correlate the obtained bioinformatic findings of FhCatL1 or FgCatL1 with those of realistic works conducted in the immunodiagnostics and preventive studies.

### Potentials of *Fasciola* spp. cathepsin L1 as a diagnostic and vaccine antigen based on previous reports

In addition to zoonotic importance, fascioliasis of livestock has a direct major financial impact globally through production losses. Such losses can include poor weight gain or productivity, condemnation of liver, lower commercial return, and inability to trade nationally and internationally (1, 2).

The accurate diagnosis of *Fasciola* infections is central to studying the epidemiology of fascioliasis and the surveillance and control of this disease. Two main species of *Fasciola* named *F. hepatica* and *F. gigantica* have been studied and showed great impact on human and animal health. Numerous studies have reported the aspects of the evaluation and validation of CatL1 antigen in diagnosis of different types of fascioliasis in various animals. In case of *F. hepatica*, recombinant CatL1 antigen (rFhCatL1)-based indirect ELISAs demonstrated high efficacy in detection of specific antibodies in infected animals including sheep (43–45). In addition, this method (rFhCatL1-based ELISA) showed excellent performance against sera of naturally or experimentally infected cattle (43, 45, 46). Also, Cornelissen et al. (47), compared indirect ELISAs of peptide and purified FhCatL1 against excreted secreted (ES) antigens of *F. hepatica* in naturally and experimentally infected cattle. This study showed a sensitivity of 98.9% for the peptide, 100% for purified FhCatL1 and 100% for ES. For specificity, values of 99.8% were found for the peptide, 94.6% for purified FhCatL1, and 82.8% for the ES antigens. Noteworthy, this study used a residue containing 15 amino acids, was predicted in our study as a sequence included in our detected epitope number 3 ranged from 58-112 of FhCatL1 used sequence. In such study, selected peptide sequence was based on immunoproteomics not on bioinformatics as in our case. An immunogenic antigen released *in vitro* by *F. hepatica* was purified. After purification the sequence of the first 20 N-terminal aa of this protein showed considerable homology with cathepsin L-like proteinase Cornelissen et al. (47). Good reactivity was observed also against cattle sera using multiplex fluorescence immunoassay (MFI) based on rFhCatL1 (48), and western blotting relying on chimeric protein of leucine aminopeptidase (LAP) and FhCatL1 (49). In the later study, FhCatL1 (GenBank CAC12806.1) that is matching and identical to our used protein (GenBank AJ279092.1) was tested, and the constructed chimeric protein included amino acid sequence from 173 to 309 which includes 6 of our identified epitopes consisting of 61 amino acids.

Moreover, rFhCatL1- ELISA was tested against naturally infected horse sera and showed high specificity of 95.6%, but low sensitivity of 42.1% compared with the reference standard diagnosis (50). Not only FhCatL1 but also *F. gigantica* cathepsin L1 (FgCatL1) antigen has proven its potential in discriminating between infected and non-infected sera collected from susceptible animals. In this regard, various forms of FgCatL1 could detect fascioliasis in buffaloes, sheep, or cattle sera either using rFgCatL1 (51), synthetic peptide (52), or monoclonal antibody against rFgCatL1 (53) based ELISAs, respectively.

Even for human sera, FhCatL1 and FgCatL1 have demonstrated an excellent ability in detecting specific antibody in infected patients with very low cross-reactivity. Many studies have utilized the indirect ELISAs based on rFhCatL1 (19, 54–60). Consistently, FgCatL1 based ELISAs showed an optimal performance against human sera when used as a recombinant protein (61, 62), or specific peptides prepared from rFgCatL1 (63, 64). Using another test different than ELISA, the lateral flow test based on rFhCatL1showed maximal specificity and sensitivity compared to ELISA either against human serum or whole blood samples (65). Recently, the recombinant antigen of cathepsin L1 prepared from *Fasciola* strain wuh2-1 (*F. hepatica/gigantica* hybrid type) showed a high efficacy in diagnosis of fascioliasis using indirect ELISAs against sera obtained from Sika deer (66), and human sera (67) from Japan.

Thus, cathepsin L1 demonstrated its potential ability as a global diagnostic antigen for the detection of fascioliasis of various *Fasciola* species, in different diagnostic tests and for multiple susceptible species. The developed tests demonstrated sensitivity from 88% to a maximum of 100% and specificity from 94.6 to 100% in highly susceptible animals like sheep, goat, and cattle. However, these values have been slightly reduced in other hosts as horse and human. The results obtained are satisfactory, and therefore, some of the tests may overcome the disadvantages of the reference technique or become an efficient screening method. However, although indirect ELISAs demonstrate efficiency in the diagnosis of fascioliasis, other tests are used less frequently. Among these, western blotting and MFI are reliable methods with a high sensitivity and specificity for antibody detection and are widely used to evaluate serological assays performances. Even for ELISA, indirect ELISA format tests are mostly used, which are both sensitive and specific. Other ELISA formats should be also developed to increase the accuracy of this technique, for example, competitive ELISA, blocking ELISA, and capture ELISA, which use mAbs or pAbs that bind to the same epitope. Moreover, these tests do not require the use of secondary antibodies, which are specific for different species, and IgM and IgG antibodies can be tested in the same assay.

Most of the tests developed in the last decade have focused mainly in detection of antibodies against *F. hepatica* rather than *F. gigantica* and in human instead of highly susceptible animals as sheep and cattle (Table 2), and only very few studies focused on the serodiagnosis of fascioliasis in other species. In order to minimize false positives, most of the work done in the last few years has used specific *Fasciola* recombinant proteins (Table 2) using different expression systems. However, the use of native ES proteins in their original conformation avoids undesirable changes that can occur in heterologous expression systems (11). However, recombinant antigens have been used to attempt to overcome the disadvantage of variation in quality and quantity of native antigens derived directly from *Fasciola*. Also, the use of purified native antigens has a high probability of cross reactivity (47). Purification of proteins may also influence the performance of the developed tests. Thus, preparation of specific peptides based on epitopes mapping and selection of unique sequences and using as single peptide or as chimeric protein using available the free or the commercial online websites or programs will improve the diagnostic procedures against fascioliasis in animals and humans.

**Table 2:** Previous diagnostic studies on *Fasciola* cathepsin L1 antigen on different animal sera.

***Fasciola* species**	**Type of antigen**	**Type of test**	**Target animals**	**Findings and data**	**References**
*Fasciola hepatica*	rFhCatL1, rFhCatL2, rFhCatL5	iELISA	Ni Cattle & Ni Sheep	Cattle, sensitivity were 97% for rFhCatL2 and 87.9% for both rFhCatL1 and rFhCatL5. In sheep, the sensitivity was 100% for all.	(43)
*Fasciola hepatica*	rFhCatL1, rFhFABP	iELISA	Ni Sheep	rFhCatL1 showed 100% sensitivity and 97% specificity, while rFhFABP was 95% for sensitivity.	(44)
*Fasciola hepatica*	rFhCatL1	iELISA	Ni Cattle, sheep & Ei Cattle, sheep	rFhCatL1 showed better performance in experimentally infected cattle and sheep sera than naturally infected ones.	(45)
*Fasciola hepatica*	rFhCatL1	iELISA	Ei Cattle	rFhCatL1 sensitivity was low during prepatency (20–85% during 4–7 wpi), but increased with early beginning of patency and reached 100% during 10–15 wpi. Then, sensitivity ranged 90% and 100%.	(46)
*Fasciola hepatica*	Peptide FhCatL1, ES FhCatL1, purified FhCatL1	iELISA	Ni & Ei Cattle	The sensitivities were peptide 98.9%, 100% ES and 100% purified FhCatL1. The specificity of the peptide ELISA was 99.8%, and was 82.8 and 94.6% for ES and FhCatL1, respectively.	(47)
*Fasciola hepatica*	rFhCatL1	M-IFA	Ei Cattle	The test showed a 100 % sensitivity and 100 % specificity.	(48)
*Fasciola hepatica*	FhCatL1, rFhLAP	Western blot	Ni Cattle	Good reactivity was observed using chimeric protein hCatL1-rFhLAP-.	(49)
*Fasciola hepatica*	rFhCatL1	iELISA	Ni Horses	rFhCatL1 showed high specificity 95.6%, but low sensitivity 42.1% compared with the reference standard diagnosis.	(50)
*Fasciola gigantica*	rFgCatL1cp, FgCatL1-D	iELISA	Ni & Ei Buffaloes	High sensitivity 97.1% and specificity 100% of rFgCatL1 cysteine proteinase and rFgCatL1-D was obtained.	(51)
*Fasciola gigantica*	Two synthetic peptides FgCatL1	iELISA	Ni Cattle & sheep	The sensitivity of peptide 1 and 2 was 100%. The specificity of peptide 1 and 2 was 87.3 and 79%, respectively.	(52)
*Fasciola gigantica*	rFgCatL1	sELISA, ICT	Ei Mouse & Ni Cattle	In mice, the sensitivity and specificity were 95% and 100% for ELISA, and 93% and 100% for ICT. In cattle, the sensitivity and specificity were 98.3 and 100% ELISA, and 96.7, and 100% for ICT, respectively.	(53)
*Fasciola hepatica*	rFhCatL1, crude ES	iELISA	Human	FhCatL1 showed sensitivity 76.9%, while ES detected 100% but with lower specificity.	(54)
*Fasciola hepatica*	rFhCatL1 native FhCatL1	iELISA	Human	A highly statistically significant correlation (r2 = 0.751, P < 0.001) was demonstrated between the absorbances of Recombinant and native FhCatL1.	(55)
*Fasciola hepatica*	rFhCatL1	iELISA	Human and Ei Sheep	The sensitivity and specificity of the rFhCatL1 were 100% in human and sheep tested sera	(56)
*Fasciola hepatica*	rFhCatL1 ES-ELISA	iELISA	Human	Both rFhCatL1 and ES ELISA exhibited a sensitivity of 100% and a specificity of 100 and 98.9%, respectively	(57)
*Fasciola hepatica*	rFhCatL1	iELISA	Human	99.9% sensitivity and 99.9% specificity was obtained.	(58)
*Fasciola hepatica*	rFhCatL1, rFhSAP2	iELISA	Human	The best diagnostic results were obtained using the rFhSAP2-ELISA (87% sensitivity, 99% specificity).	(59)
*Fasciola hepatica*	rFhCatL1, rFhFABP, rGST	iELISA	Human	rFhCatL1 was more sensitive and specific (100%) than the others.	(60)
*Fasciola hepatica*	FhMEP	iELISA	Human	The rMEP was highly antigenic, and immune-detection techniques revealed the specificity of antigen.	(19)
*Fasciola gigantica*	rFgCatL1	iELISA	Human	The sensitivity, specificity, accuracy, positive predictive value, and negative predictive value of rFgCatL1 ELISA were 100, 98.92, 98.97, 81.25, and 100%, respectively.	(61)
*Fasciola gigantica*	rFgCatL1	iELISA	Human	The sensitivity, specificity, accuracy, and positive and negative predictive values of the test IgG4 ELISA were 100%, 99.3%, 99.3%, 86.7%, and 100%, respectively.	(62)
*Fasciola gigantica*	Two peptide- rFgCatL1, crude ES	iELISA	Human	The sensitivity, specificity, accuracy, and positive and negative predictive values of this peptide-based ELISA with both peptides were 100, 97.3, 97.5, 61.9, and 100%, respectively.	(63)
*Fasciola gigantica*	Two peptide- rFgCatL1	iELISA	Human	The sensitivity, specificity, accuracy, and positive and negative predictive values of this assay were the same with both peptides at 100, 99.7, 99.7, 96.2, and 100%, respectively.	(64)
*Fasciola hepatica*	rFhCatL1	LFI	Human	Comparing to ELISA test (MM3-SERO) LFI showed maximal specificity and sensitivity and can be used.	(65)
*Fasciola* wuh2-1 (F. *hepatica/ gigantica* hybrid type)	rFwCatL1	iELISA	Ni Sika deer	The sensitivity and specificity of the ELISA were 84.6% and 100%, respectively.	(66)
*Fasciola* wuh2-1 (F. *hepatica/ gigantica* hybrid type)	rFwCatL1	iELISA	Human	The rFhCatL1-ELISA showed 100% for the sensitivity and specificity against the control samples. In test sera, the sensitivity and specificity was 100% and 99.0%, respectively.	(67)

Ni, naturally infected; Ei, experimentally infected; iELISA, indirect ELISA; sELISA, sandwich ELISA; ICT, immunochromatographic test; MEP, The tertiary structures of rFhCatL1, saposin-like protein 2 and 16.5-kDa tegument-associated protein were used as multi-epitopes protein (MEP); rFhFABP, fatty acid binding protein; M-IFA, Multiplex fluorescence immunoassay; LAP, Chimeric protein of leucine aminopeptidase; rFgCatL1cp, cysteine proteinase; rFhSAP2, saposin-like protein-2; rGST; glutathione-S-transferase; LFI, Lateral flow immunoassay.

Similarly, numerous studies have assessed FhCatL1 and FgCatL1 as potential vaccine candidates using different animal models (Table 3). In this regard, several studies have demonstrated the immunoprotective properties of mimotopes derived from FhCatL1: Mimotopes are peptides mimicking protein that can be generated by phage display technology. This strategy showed promising results in vaccinated sheep as evidenced in reduced fluke burden and egg output combined with elicited protective immune response (68–71). Similar results were obtained when goats (72, 73), and cattle (74) were used as a model for vaccination using FhCatL1 mimotopes.

**Table 3:** Previous studies on *Fasciola* cathepsin L1 as a vaccine antigen in different animal models.

***Fasciola* species**	**Type of vaccine antigen**	**Model**	**Main findings in vaccinated vs. non-vaccinated**	**References**
*Fasciola hepatica*	pFhCatL1/2 (Mimotopes)	Sheep	Reduced worm burdens, worm size, and egg output.	(68)
*Fasciola hepatica*	pFhCatL1 (Mimotopes)	Sheep	Reduced fluke burden, fluke length and width, wet weights and egg output.	(69)
*Fasciola hepatica*	pFhCatL1 (Mimotopes)	Sheep	Vaccination with pFhCatL1 in single or in combined form reduces size, fluke burden, egg production and viability in vaccinated animals.	(70)
*Fasciola hepatica*	pFhCatL1 (Mimotopes), ES	Sheep	Induction of protective cellular and humoral immune responses.	(71)
*Fasciola hepatica*	pFhCatL1 (Mimotopes) with Quil A and wild-type M13KE phage	Goat	Reduced worm burdens, and eggs output.	(72)
*Fasciola hepatica*	pFhCatL1 (Mimotopes)	Goats	Reduced worm burden, morphometric measurements, and reproductive structures	(73)
*Fasciola hepatica*	p FhCL1 protein	Cattle	Reduced fluke burden, and induction of specific antibody responses.	(74)
*Fasciola hepatica*	rFhCatL1 plus Quil A	Goat	Reduced hepatic damage.	(75)
*Fasciola hepatica*	rFhCatL1, peroxiredoxin and Sm14 antigen	Goat	Induction of specific antibody responses	(76)
*Fasciola hepatica*	rFhCatL1 with or without Quil A	Goat	Showed higher weight gain and reduced severity of hepatic lesions.	(77)
*Fasciola hepatica*	rFhCatL1 and hemoglobin	Cattle	Reduced fluke burden either in single or combined with Hb.	(10)
*Fasciola hepatica*	A combination of FhCatL1, FhCatL2 or FhCatL2 and FhFHB	Cattle	Reduced fluke burdens, with highest protection in group vaccinated with FhCatL1 plus FhCatL2	(78)
*Fasciola hepatica*	rFhCatL1 in Montanide ISA 70VG and ISA 206VG.	Cattle	Reduced fluke burden in the cattle in both vaccinated groups.	(79)
*Fasciola hepatica*	rFhCatL1cp	Rat	Increased protection 78-80% against challenge with metacercariae. CD8+ and CD4+ lymphocytes were higher numbers in the peritoneal fluid.	(80)
*Fasciola hepatica*	CiFhCatL1, FhLAP	Rabbit	Induction of strong specific antibody responses.	(49)
*Fasciola hepatica*	CiFhCatL1 and FhLAP	Sheep	Slight reduction of fluke burden and egg output.	(81)
*Fasciola gigantica*	Pro and mature rFgCatL1 with Freund's adjuvant.	Mice	Reduced liver damage and induction of specific antibody response	(82)
*Fasciola gigantica*	Pro rFgCatL1H	Mice	Reduced infection rate and induction of specific antibody response.	(83)

pFhCatL1, peptide of Fasciola hepatica cathepsin L1; rFhCatL1, recombinant protein of FhCatL1; CiFhCatL1, chimeric protein of FhCatL1; FhFHB, fluke-derived Hb fraction; rFgCatL1cp, cysteine proteinase recombinant protein of FhCatL1; FhLAP, leucine aminopeptidase.

Furthermore, rFhCatL1 alone or formulated with Quil A adjuvant as a different approach of vaccination showed partial protection manifested in reduced liver changes and increased body gains in immunized goats (75–77) compared to the control non-immunized groups. Similar tendency was also reported when cattle was immunized with rFhCatL1 (10, 78, 79). Even in rat, enteral vaccination with rFhCatL1 induced high protection against challenge with fluke metacercariae (80).

Other studies have reported the usefulness of chimeric vaccine antigen prepared from FhCatL1 and leucine aminopeptidase (FhLAP) in eliciting an immune response and conferring protection against challenge infection in both rabbit (49), and sheep (81). Concerning vaccine researches applied on *F. gigantica*, immunization of mice with recombinant pro and mature FgCatL1 showed a protective potential against challenge infection when compared with non-immunized control group (82, 83).

These results revealed the usefulness of cathepsin L1 as a vaccine antigen against ovine, caprine, bovine and murine fascioliasis. Vaccines can be used to prevent, manage, or eradicate disease and are set to become increasingly important as front-line control tools, especially as many parasites including *Fasciola* progressively emerge with wide resistance to available anti-parasitic drugs and the burden of parasites. Different preparations of FhCatL1 including recombinant protein or mimotope and either used solitary or in combination with other antigens succeeded to confer efficient protective potentials in different animal models. However, further studies are required to improve the protective potentials, eg. by mixing with potent adjuvants, testing the underestimated *F. gigantica*, and finding out novel protective indices. As the vaccine preparation and testing are costly procedures, initial assessment of the efficacy will be highly advantageous in many aspects particularly from economic viewpoint. Bioinformatic analysis has been already providing this benefit for vaccine researches including those conducted for *Fasciola* assessments (20). The identification of potent immuno-epitopes from cathepsin L1 from *F. hepatica* or *F. gigantica* using our presented analyses software might contribute in improvement of vaccine efficacy *via* wet-lab approach not only the theoretical aspect. This could be proven via conducting certain preliminary experiments based on the interaction of a prepared antigen with immune-effectors cells (macrophages, dendritic cells, Treg cells) or molecules (Interleukin β1, IL-10, MHC classes I and II proteins) (84, 85). Currently, the selection of the targeted cell or molecule can be determined using a protein or peptide of known sequence *via* the available online websites and programs software.

## Conclusion

We revealed the first description of multiple sequence alignment, isoelectric point, phosphorylation sites, and morpho-physical properties from protein database of FhCatL1. Moreover, first assessment of FhCatL1 was applied for secondary structure using PSIPRED and GOR IV servers, tertiary, and quaternary structure using SWISS-MODEL, and linear B- cell epitopes using ProtScale online available tool. The bioinformatic analyses in the present study revealed the high antigenicity, immunogenicity, hydrophilicity, surface accessibility, and flexibility indexes for FhCatL1 protein. Thus, application of on-line available bioinformatic analysis tools can be used for evaluation and validation of basic structures and properties and to predict antigenicity and immunogenicity of protein based on amino acid sequence. Such data are additionally supported with numerous previous literatures that corroborating the high efficiency of FhCatL1 as diagnostic and vaccine antigen. Hence, we recommend FhCatL1 as a suitable antigen for developing diagnostic tests and vaccines against fascioliasis in different animal models. Prediction of the antigenic or immunogenic properties of *Fasciola-* or other parasites-derived proteins *via* computational and online software programs will reduce the time, effort and cost before application of practical assays and experiments. Useful relevant information was also presented for FgCatL1 as a diagnostic and vaccine candidates, although it was much less than those of FhCatL1.

## Data availability statement

The datasets presented in this study can be found in online repositories. The names of the repository/repositories and accession number(s) can be found in the article/Supplementary material.

## Author contributions

Conceptualization and investigation: RF and AA. Formal analysis, project administration, and writing—original draft: RF. Methodology: RF, SM, E-SE-A, HA, and MO. Resources and writing—review and editing: RF, SM, E-SE-A, HA, OS, MO, and AA. All authors contributed to the article and approved the submitted version.
