# Supplementary material for: High relatedness of bioinformatic data and realistic experimental works on the potentials of Fasciola hepatica and F. gigantica cathepsin L1 as a diagnostic and vaccine antigen
Source: Front Public Health. 2022 Dec 7;10:1054502. doi: 10.3389/fpubh.2022.1054502 (PMC9768368; doi:10.3389/fpubh.2022.1054502)
Supplement: Supplementary file 1 [file Data_Sheet_1.PDF]

Supplemental information

Table S1. Summary of the physicochemical and topological properties of *F. hepatica* and *F. gigantica*

Item	Test / tool	*F. hepatica*	*F. gigantica*	Notes
Molecular weight	IPC	37.32	37.32	Strong immunogen
Diameter		4.67487 nm	4.67487 nm	Highly interactive
Isoelectric point		5.2	5.11	Essential for analytical chemistry
Multiple sequence alignment	CLUSTAL OMEGA	94.9% vs each other		Moderate similarity to human and cattle cathepsin L1.
Signal peptide cleavage sites	SignalP 5.0	No	No	This protein sequence is lacking the first 23 amino acids,
Transmembrane helices in proteins	TMHMM 2.0c	No	No	
Phosphorylation sites	NetPhos 3.1	33 sites	33 sites	High liability of protein for PTM
Secondary structure	PSIPRED & GOR IV	53.38 % random coil, 24.44 % extended strand, and 22.19 % alpha helix	52.41 % random coil, 25.08 % extended strand, and 22.51 % alpha helix	High stability
Tertiary structure	ExPasy Swiss model (Coverage)	1.00	1.00	High protein modeling
	Ramachandran Plots	Ramachandran favored (95.78%), no Ramachandran	Ramachandran favored (96.10%), Ramachandran	

		outliers, C-Beta deviations or bad bonds, Rotamer outliers (1.16%), bad angels (30 / 6856) and twisted proline (2/18)	outliers 0.32%, C-Beta deviations (2) bad bonds (0/5062), Rotamer outliers (1.15%), bad angels (34 / 6858) and twisted proline (2/18)	
	ExPasy Protscale model	Strong signals were obtained in all the plots of hydrophobicity, alpha-helix, beta-turn, average flexi-bility, percent of accessible residue, and molecular weight	Strong signals were obtained in all the plots of hydrophobicity, alpha-helix, beta-turn, average flexi-bility, percent of accessible residue, and molecular weight	High liability of presence of linear B-cell epitopes
Quaternary structure	SWISS-MODEL	High consistency of PPI fingerprint with deposited model 206X in PDB and SWISS-MODEL	-	Oligomeric state, stoichiometry, topology and interface similarity were assessed
Hydrophilicity	Parker hydrophilicity prediction	Threshold 1.877	Threshold 1.912	High index of water solubility

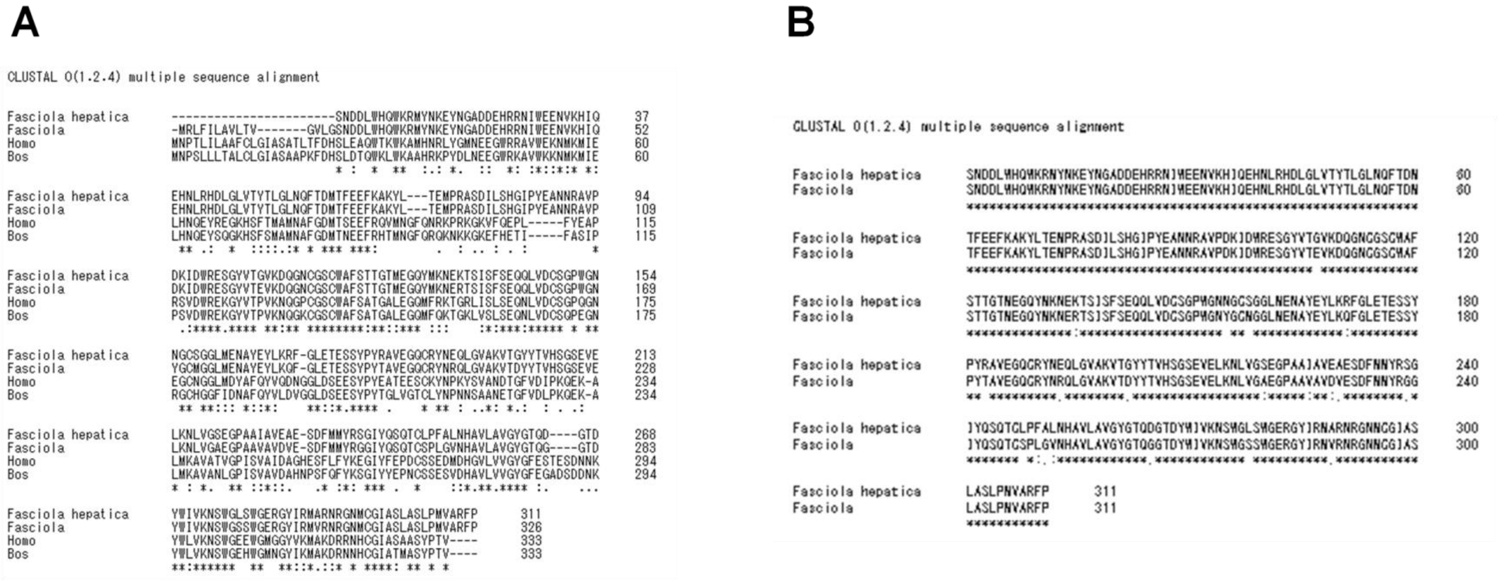

Figure S1. Sequence alignment of FhCatL1 amino acids. **(A)** Multiple sequence alignment of FhCatL1 was applied by CLUSTALO against amino acid sequences of *F. gigantica*, human and cattle. Similarity was 94.2%, 44.4% and 40.2% for cathepsin L1 of *F. gigantica (Fasciola)*, human (Homo) and cattle (Bos), respectively. highlighted sequences show additional amino acid sequences lacking in FhCatL1. **(B)** Sequence alignment of FhCatL1 and of FgCatL1 after removal of additional amino acid sequence in of FgCatL1 for better visualization of matching (https://www.ebi.ac.uk/Tools/msa/clustalo/).

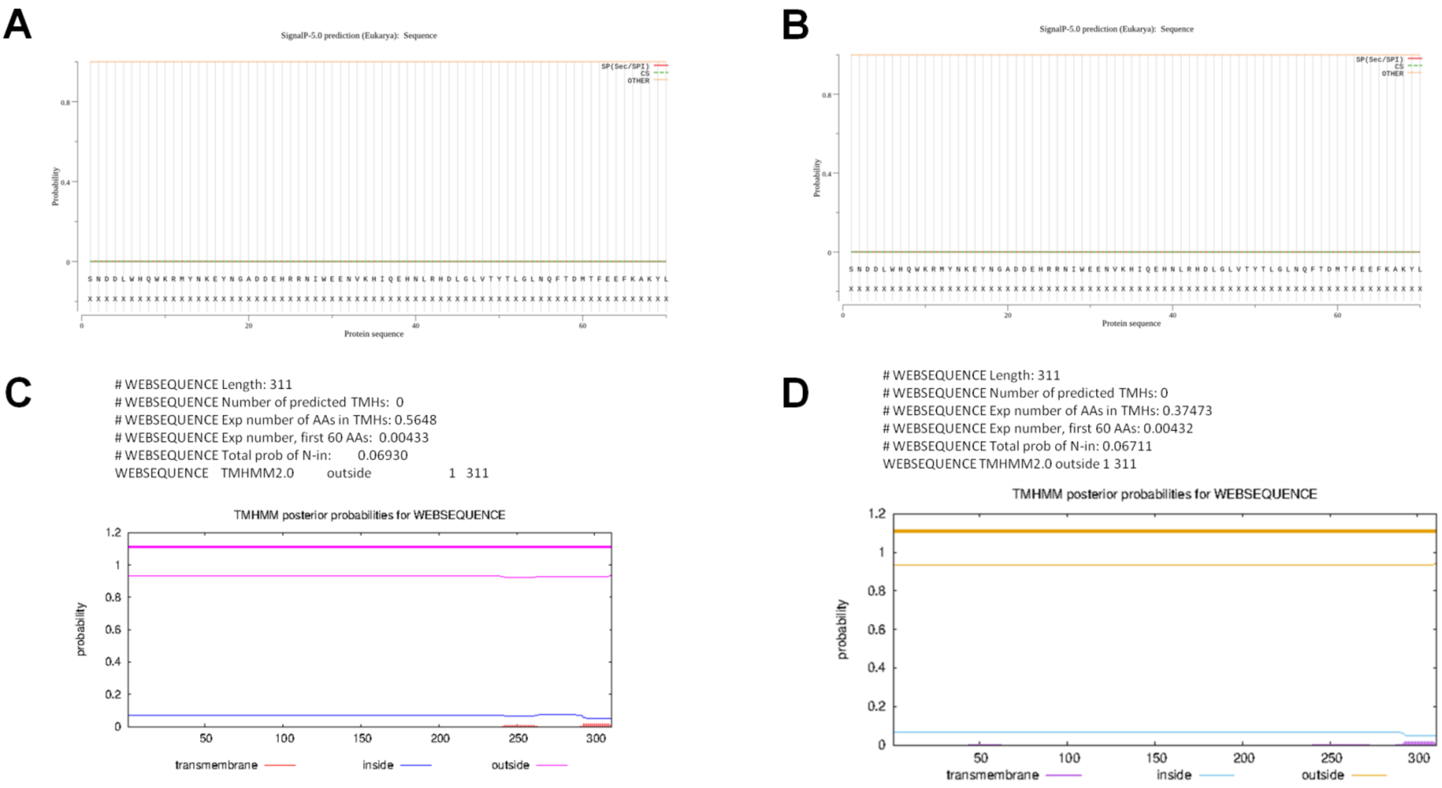

Figure S2. Signal peptide and transmembrane domains of FhCatL1 and FgCatL1 amino acids Signal peptide prediction of the FhCatL1 **(A)** and FgCatL1 **(B)** protein using SignalP-5.0 online tool. SP (Sec/SPI): type of signal peptide predicted; CS: the cleavage site; Other: the probability that the sequence does not have any kind of signal peptide (https://services.healthtech.dtu.dk/service.php?SignalP-5.0). Transmembrane domains expected in FhCatL1 **(C)** and FgCatL1 **(B)** protein. Some statistics and a list of the location of the predicted transmembrane helices and the predicted location of the intervening loop regions. Length: the length of the protein sequence; number of predicted TMHs: the number of predicted transmembrane helices; Exp number of AAs in TMHs: the expected number of amino acids in transmembrane helices. If this number is larger than 18, it is very likely to be a transmembrane protein (or have a signal peptide); Exp number, first 60 AAs: the expected number of amino acids in transmembrane helices in the first 60 amino acids of the protein. If this number is more than a few, you should be warned that a predicted transmembrane helix in the N-term could be a signal peptide; total prob of N-in: the total probability that the N-term is on the cytoplasmic side of the membrane (https://services.healthtech.dtu.dk/service.php?TMHMM-2.0).

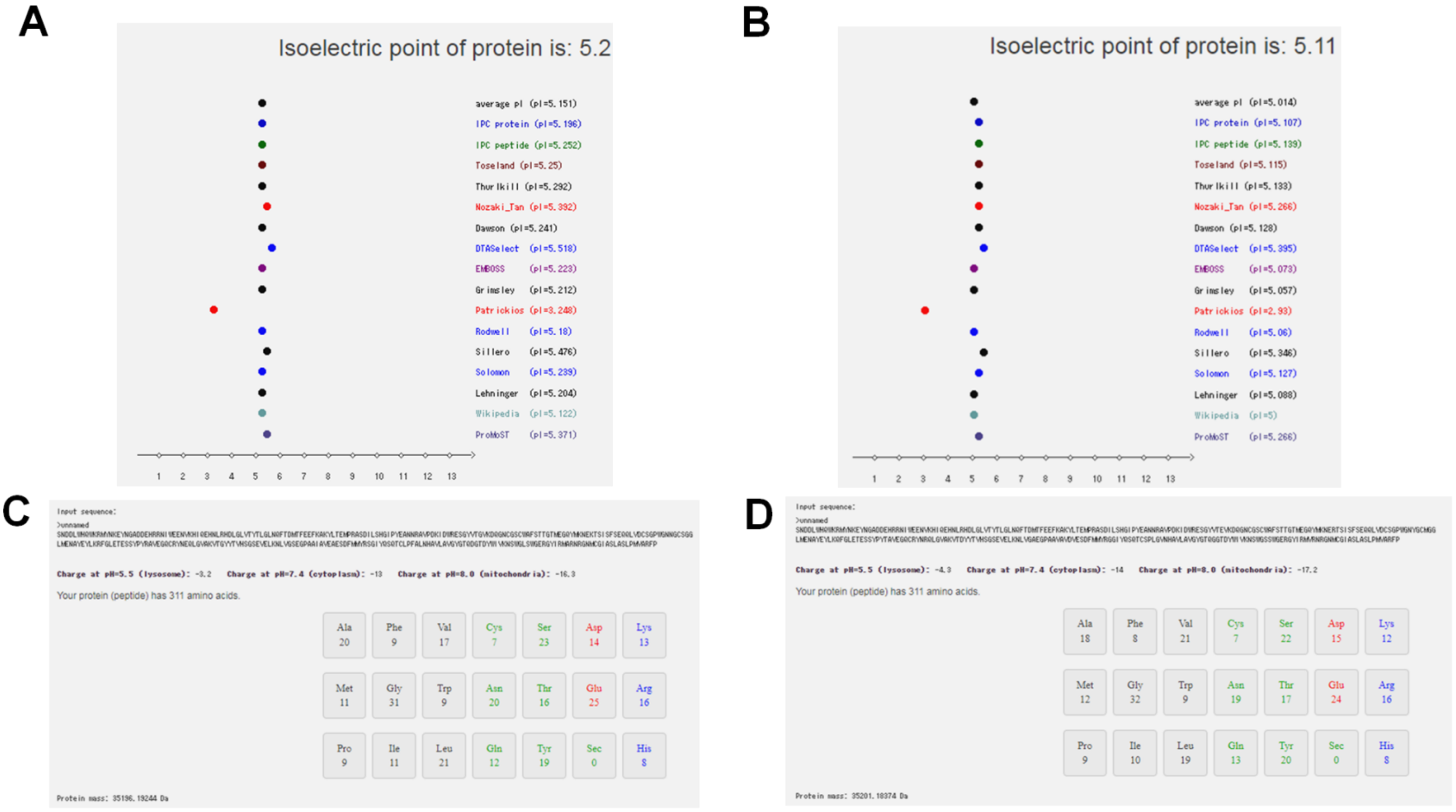

Figure S3. Isoelectric point and molecular mass of FhCatL1 and FgCatL1 amino acids. The theoretical isoelectric point pI of FhCatL1 **(A)** and FgCatL1 **(B)** showing average and different predictors values (http://isoelectric.org/). The molecular mass and amino acids sequence patterns in FhCatL1 **(C)** and FgCatL1 **(D)** (http://isoelectric.org/). Results showed very similar results concerning calculated pI, molecular mass and amino acid structures.

A

```
..
     SNDDLWHQWKRMYNKEYNGADDEHRRNIWEENVKHIQEHNLRHDLGLVTY   #   50
     TLGLNQFTDMTFEEFKAKYLTEMPRASDILSHGIPYEANNRAVPDKIDWR   #  100
     ESGYVTEVKDQGNCGSCWAFSTTGTMEGQYMKNERTSISFSEQQLVDCSG   #  150
     PWGNYGCMGGLMENAYEYLKQFGLETESSYPYTAVEGQCRYNRQLGVAKV   #  200
     TDYYTVHSGSEVELKNLVGAEGPAAVAVDVESDFMMYRGGIYQSQTCSPL   #  250
     GVNHAVLAVGYGTQGGTDYWIVKNSWGSSWGERGYIRMVRNRGNMCGIAS   #  300
     LASLPMVARFP                                          #  350
%1   ............Y.....................................   #   50
%1   .......T..T...............S...S....Y..............   #  100
%1   .S.Y...........S.............Y......S.S.S.........   #  150
%1   ....Y..........Y.Y.........S.Y.Y..................   #  200
%1   ..YY...S.S.....................S.........Y.S...S..   #  250
%1   ............T.....Y.....S...S.....Y..............S   #  300
%1   ...........
```

B

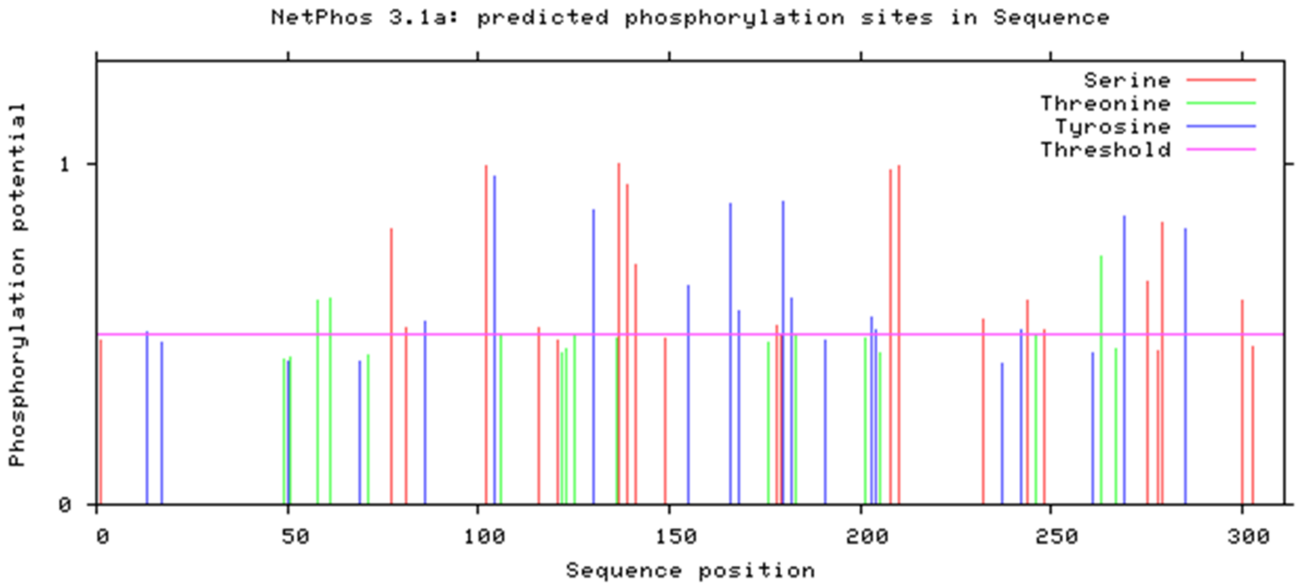

Figure S4. NetPhos server output for FgCatL1 phosphorylation sites. **(A)** The number of predicted sites, based on S (serine), T (threonine) and Y (tyrosine); **(B)** Prediction diagram of FgCatL1 phosphorylation sites (https://services.healthtech.dtu.dk/service.php?NetPhos-3.1).

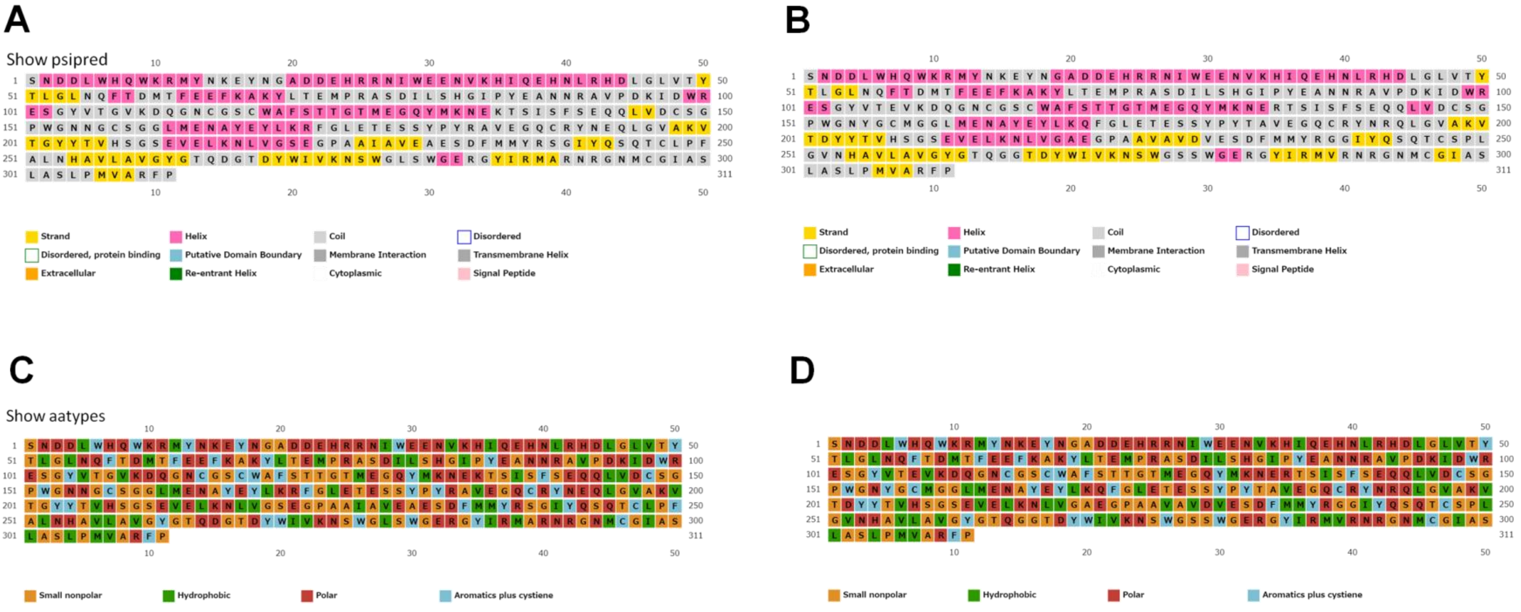

Figure S5. Graphical output of secondary structure of FhCatL1 and FgCatL1. Illustration of the secondary structure prediction using PSI-BLAST (http://bioinf.cs.ucl.ac.uk/psipred) showing psipred of FhCatL1 **(A)** and FgCatL1 **(B),** and amino acids types of FhCatL1 **(C)** and FgCatL1 **(D).**

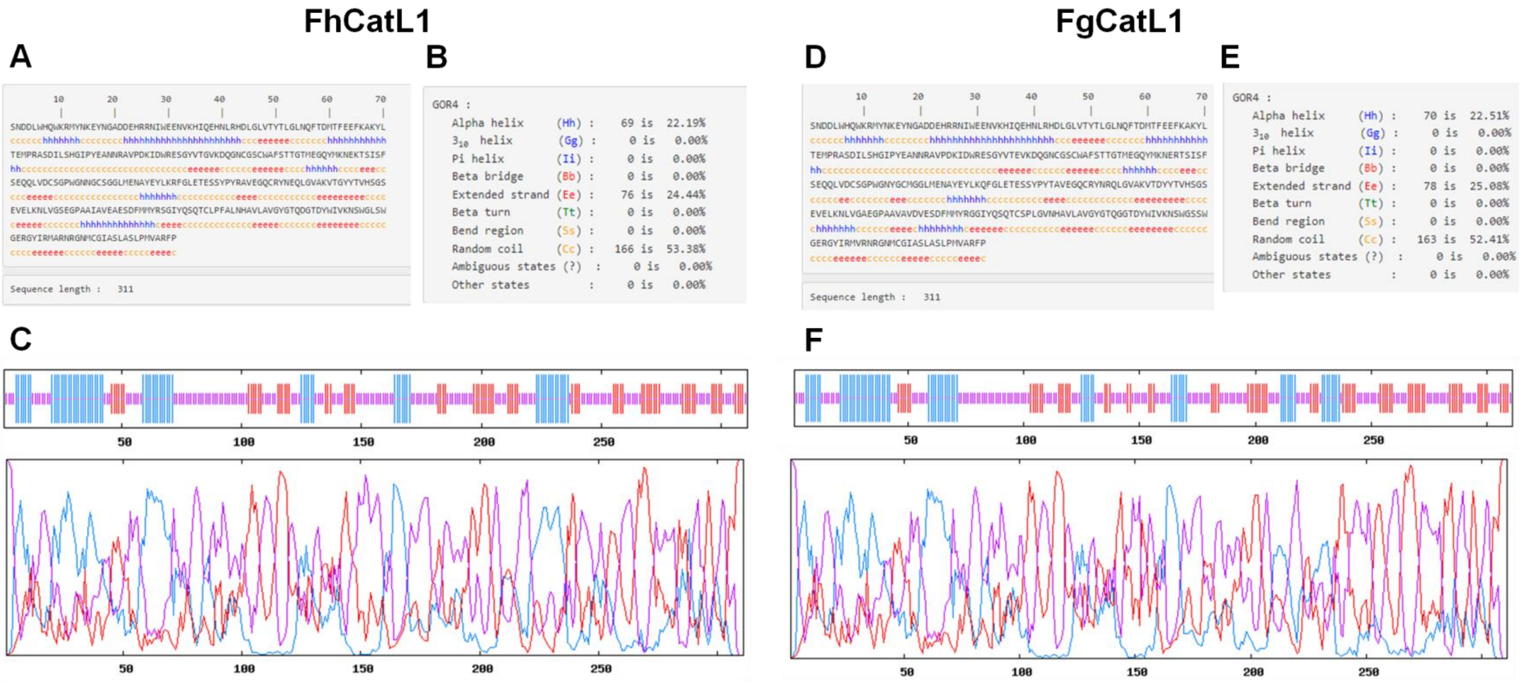

Figure S6. The secondary structure prediction of FhCatL1 and FgCatL1 based on GOR IV server. The results of FhCatL1 that showed an illustration for the type of secondary structure based on amino acid sequences **(A)**, and the percentage of different structure that encompasses 53.38 % random coil, 24.44 % extended strand, and 22.19 % α-helix in secondary structure **(B)**. **(C)** Graphical result of the secondary structure prediction of FhCatL1 using GOR IV online server. For FgCatL1 an illustration for the type of secondary structure based on amino acid sequences **(D)**, and the percentage of different structure that encompasses 52.41 % random coil, 25.08 % extended strand, and 22.51 % α-helix in secondary structure **(E)**. **(C)** Graphical result of the secondary structure prediction of FgCatL1 using GOR IV online server (https://npsa-prabi.ibcp.fr/cgi-bin/secpred_gor4.pl).

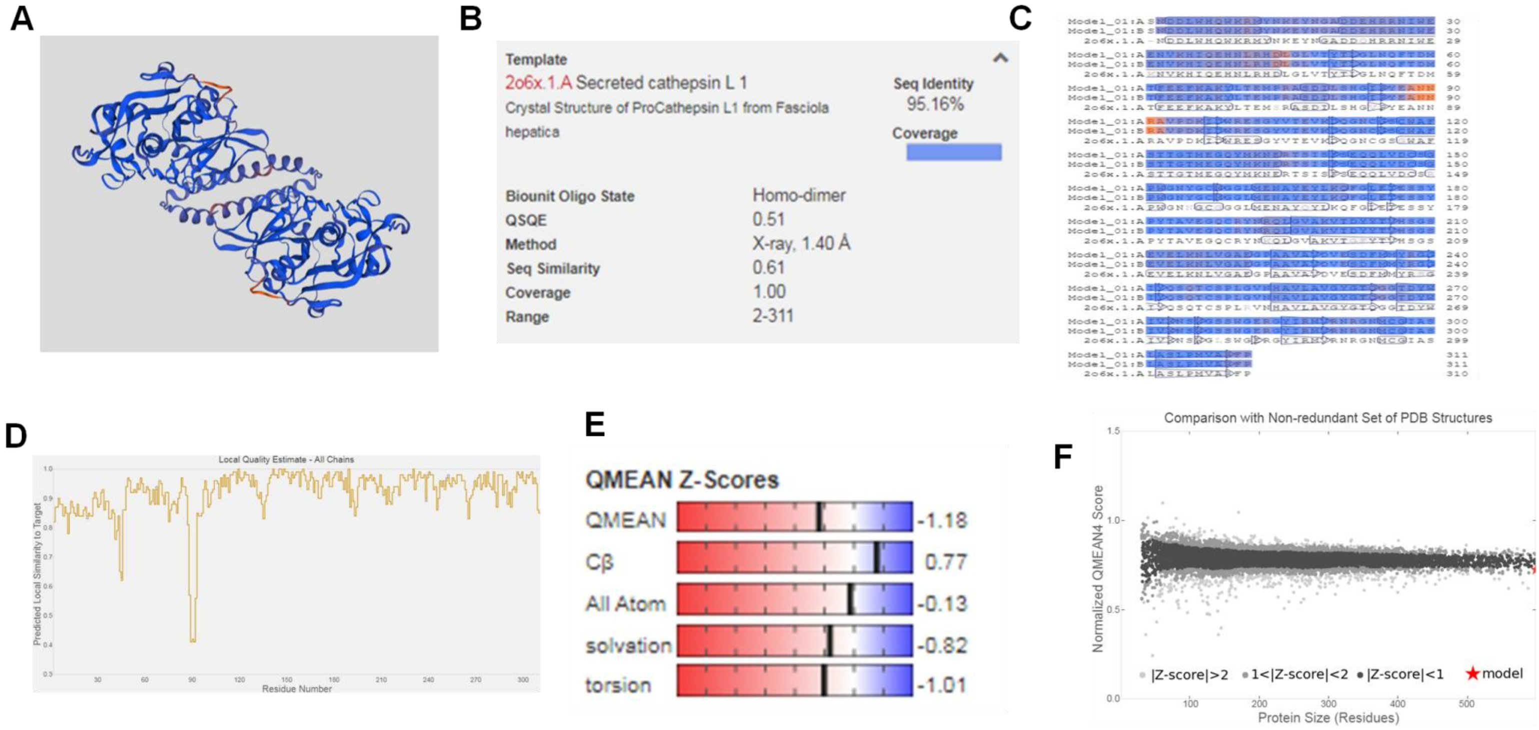

Figure S7. Tertiary structure of FGCatL1 using SWISS-MODEL server. SWISS-MODEL server results of **(A)** Computed three-dimensional model; **(B)** Sequence identity and coverage data**; (C)** Model-template alignment; **(D)** Local quality estimate; **(E)** Global quality estimate; **(F)** Comparison with non-redundant set of PDB structures (https://swissmodel.expasy.org/).

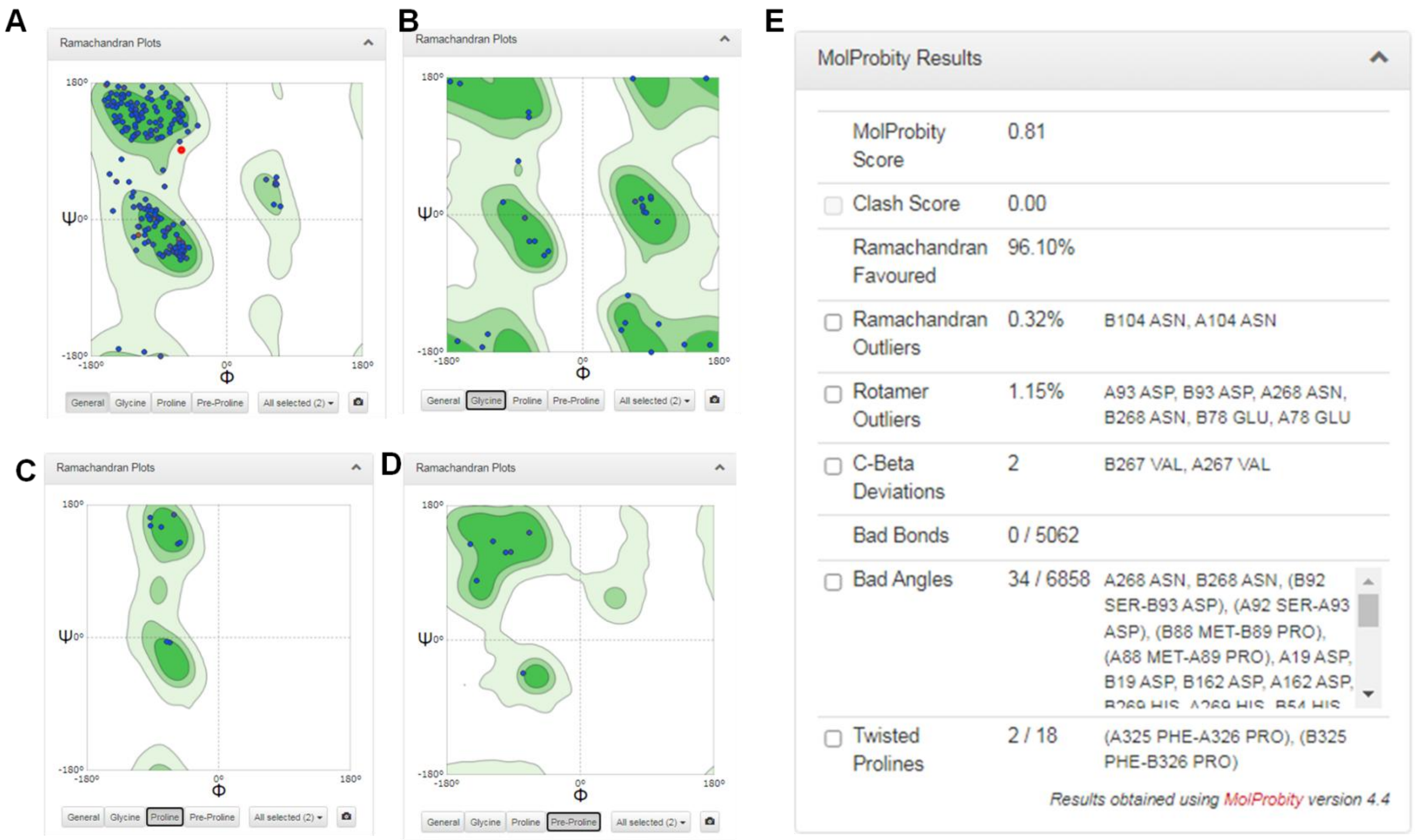

Figure S8. Analysis of 3D model of FgCatL1 via Ramachandran plot from SWISS-MODEL server. The model demonstrated MolProbity (0.81), Clash score (0.0), Ramachandran favored (96.10%), Ramachandran outliers (0.32%), C-Beta deviations (2), or bad bonds (0/5062), Rotamer outliers (1.15%), bad angels (34 / 6858) and twisted proline (2/18). **(A)** General plot, **(B)** Glycine based plot, **(C)** Proline based plot**,** **(D)** PreProline based plot, **(E)** MolProbity results (https://swissmodel.expasy.org/).

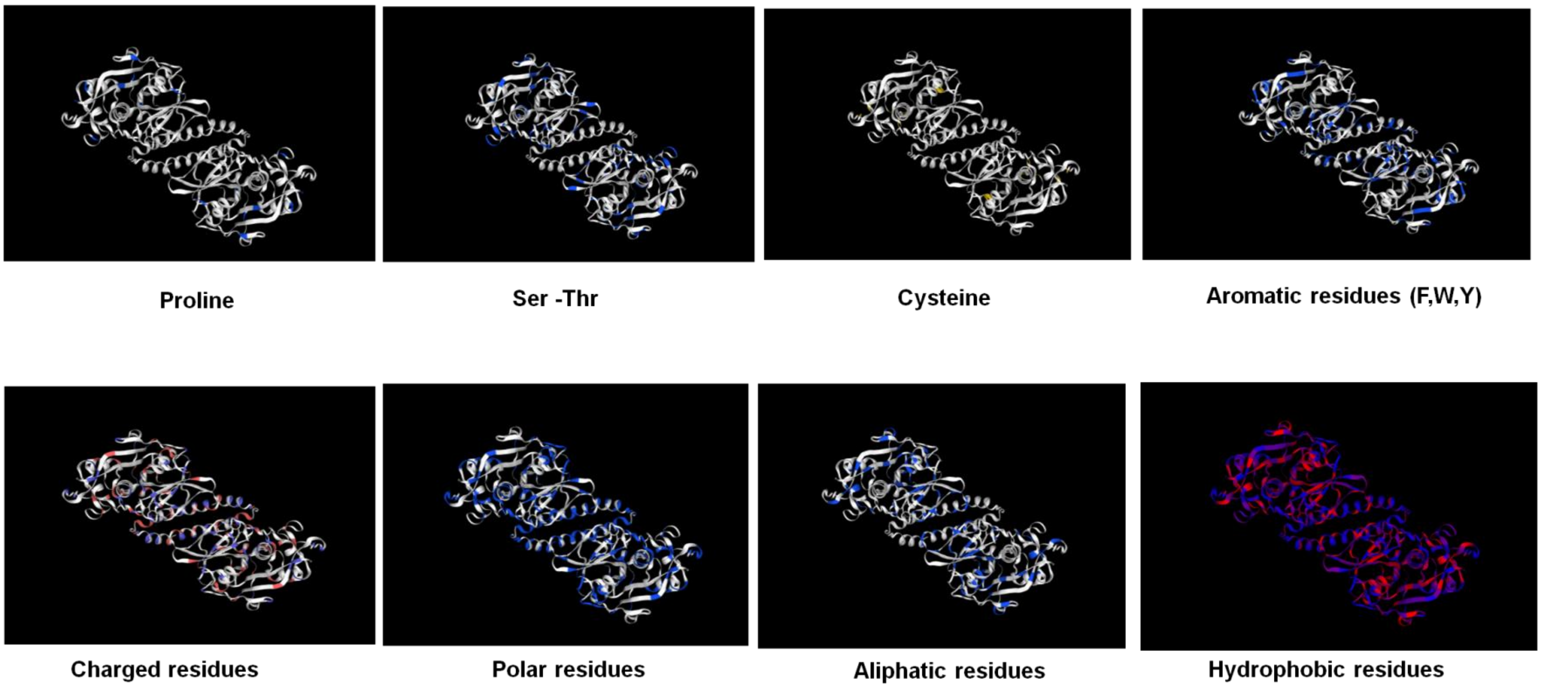

Figure S9. Mapping of different structure of amino acid residues in 3D structure model of FgCatL1 (https://swissmodel.expasy.org/).

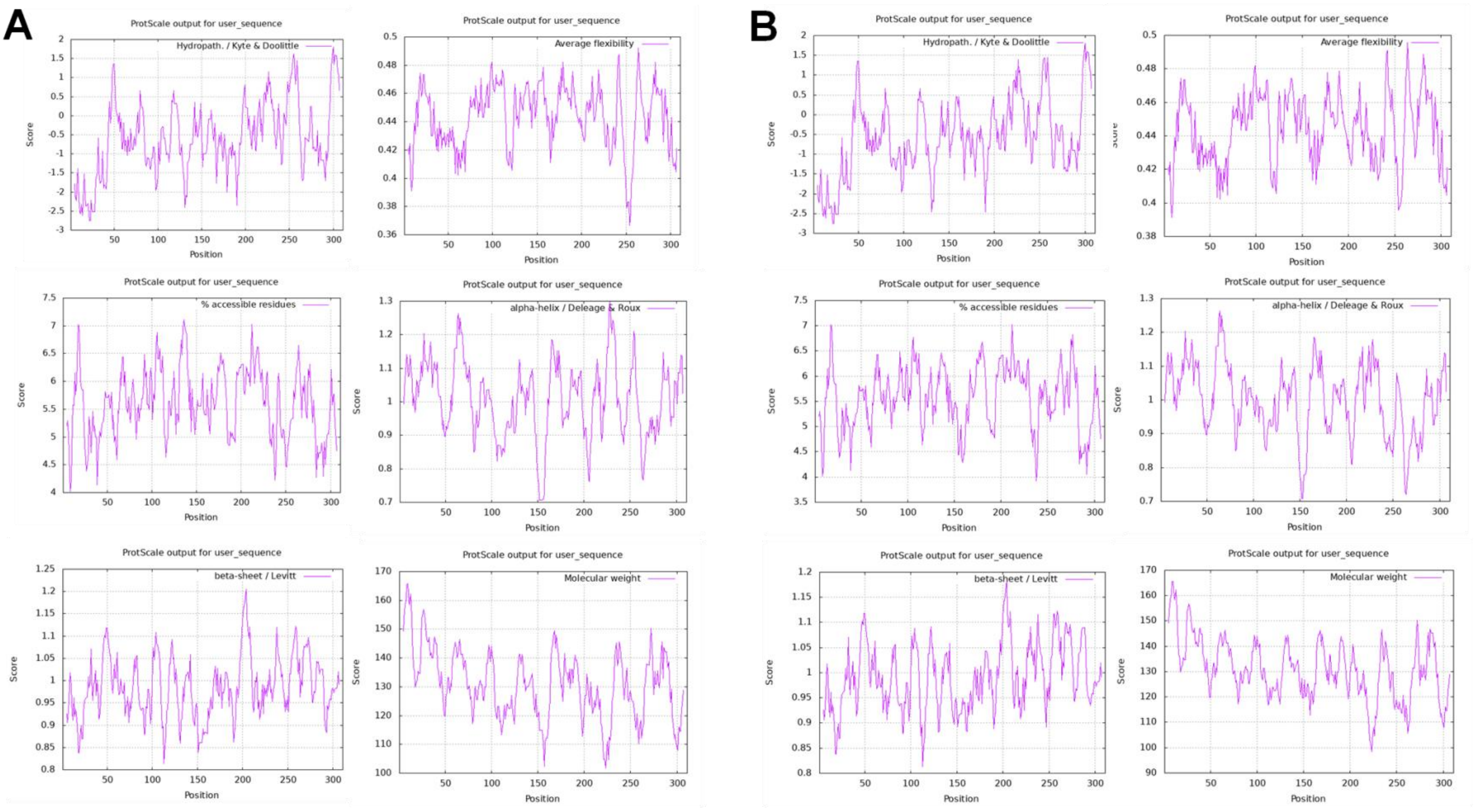

Figure S10. Linear B-cell epitopes predicted by ProtScale server (https://web.expasy.org/protscale/), for FhCatL1 **(A)** and FgCatL1 **(B)** protein sequence based on hydrophobicity, average flexibility, percent of accessible residues, Alpha helix, Beta turn, and molecular weight.

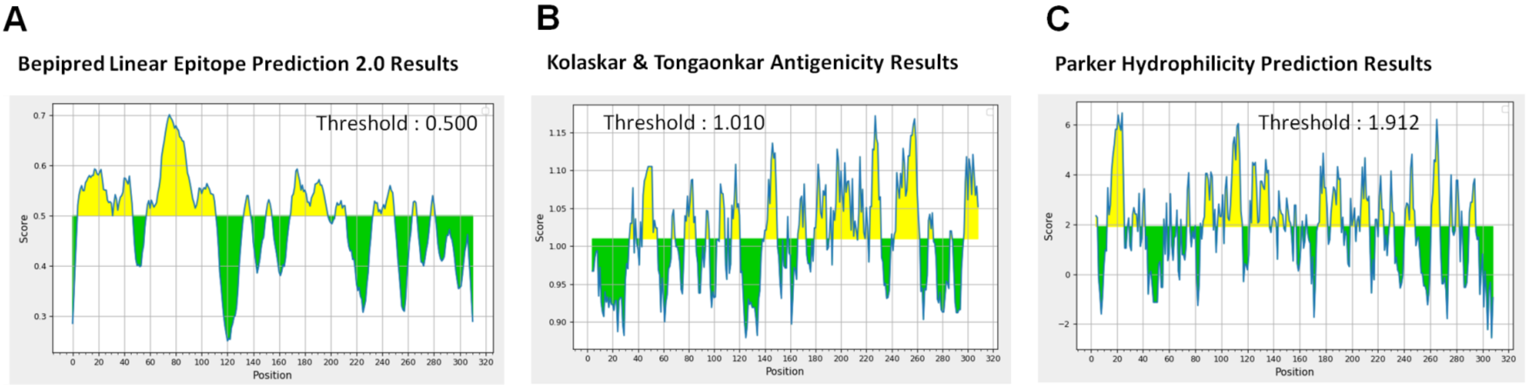

Predicted peptides:

No.	Start	End	Peptide	Length
1	5	47	LWHQWKRMYNKEYNGADDEHRRNIWEENVKHIQEHNLRHDLGL	43
2	58	112	TDMTFEEFKAKYLTEMPRASDILSHGIPYEANNRAVPDKIDWRESGYVTEVKDQG	55
3	134	139	ERTSIS	6
4	151	155	PWGNY	5
5	170	198	KQFGLETESSYPYTAVEGQCRYNRQLGVA	29
6	205	212	TVHSGSEV	8
7	234	250	FMMYRGGIYQSQTCSPL	17
8	265	267	GGT	3
9	279	282	SWGE	4

Predicted peptides:

No.	Start	End	Peptide	Length
1	44	55	DLGLVTYTLGLN	12
2	77	85	SDILSHGIP	9
3	114	120	CGSCWAF	7
4	140	149	FSEQQLVDCS	10
5	166	172	YEYLKQF	7
6	179	188	SYPYTAVEGQ	10
7	194	212	QLGVAKVTDYYTVHSGSEV	19
8	214	221	LKNLVGAE	8
9	223	231	PAAVAVDVE	9
10	242	262	YQSQTCSPLGVNHAVLAVGYG	21

Figure S11. Immunogenicity, antigenicity, and hydrophilicity of FgCatL1 protein. **(A)** Bepipred linear epitopes prediction; **(B)** Antigenicity; **(C)** Hydrophilicity. *x*-axis and *y*-axis represent position and score, respectively. The horizontal line indicates the threshold or the average score. Yellow colors (above the threshold) indicate favorable regions related to the properties of interest. Green color (below the threshold) indicates the unfavorable regions related to the properties of interest. The results were analyzed by the Immune Epitope Database (IEDB) (http://tools.immuneepitope.org/bcell/).
